# Evaluating real-world deployment of an HL7-CDA-aligned LLM for ICD-10-CM coding

**DOI:** 10.1038/s41746-026-02541-5

**Published:** 2026-04-14

**Authors:** Hong-Jie Dai, Zheng-Hao Li, An-Tai Lu, Min-I Su, Bo-Tsz Shain, Ming-Ta Li, Li-Li Wang, Kuang-Te Wang, Pei-Kang Liu, Vu Thinh Doan, Ming-Ju Tsai

**Affiliations:** 1 Department of Electrical Engineering, Intelligent System Lab, College of Electrical Engineering and Computer Science, National Kaohsiung University of Science and Technology, Kaohsiung, Taiwan, ROC; 2 National Institute of Cancer Research, National Health Research Institutes, Tainan, Taiwan, ROC; 3 Center for Big Data Research, Kaohsiung Medical University, Kaohsiung, Taiwan, ROC; 4 School of Post-Baccalaureate Medicine, Kaohsiung Medical University, Kaohsiung, Taiwan, ROC; 5 Department of Medical Records, Kaohsiung Medical University Hospital, Kaohsiung Medical University, Kaohsiung, Taiwan, ROC; 6 Division of Cardiology, Department of Internal Medicine, Taitung MacKay Memorial Hospital, Taitung, Taiwan, ROC; 7 Department of Medicine, MacKay Medical University, New Taipei, Taiwan, ROC; 8 Medical AI Innovation and Application Center, Kaohsiung Medical University Hospital, Kaohsiung Medical University, Kaohsiung, Taiwan, ROC; 9 Department of Ophthalmology, Kaohsiung Medical University Hospital, Kaohsiung Medical University, Kaohsiung, Taiwan, ROC; 10 School of Medicine, College of Medicine, Kaohsiung Medical University, Kaohsiung, Taiwan, ROC; 11 Nha Trang University, Khanh Hoa, Vietnam; 12 Division of Pulmonary and Critical Care Medicine, Department of Internal Medicine, Kaohsiung Medical University Hospital, Kaohsiung Medical University, Kaohsiung, Taiwan, ROC; 13 Department of Internal Medicine, School of Medicine, College of Medicine, Kaohsiung Medical University, Kaohsiung, Taiwan, ROC

**Keywords:** Business and industry, Computational biology and bioinformatics, Health care, Mathematics and computing, Medical research, Scientific community

## Abstract

Reliable ICD-10-CM coding remains a major operational burden in hospitals, and the real-world performance of AI systems for this task is poorly understood. We developed and deployed a modular, clinically grounded pipeline that combines principled base-model selection, redundancy-aware training, and HL7-aligned section prompts to support scalable ICD-10-CM coding across heterogeneous documentation environments. Using pairwise LLM-as-judge evaluation and Plackett–Luce ranking, BioMistral was identified as a high-performing foundation model and demonstrated consistent performance across two institutions. In a 13-week human-in-the-loop randomized controlled trial involving ten certified coding specialists, AI-assisted workflows significantly reduced coding time while maintaining accuracy. Satisfaction varied by experience, certification, and generational cohort, underscoring the importance of human factors in workflow integration. Importantly, our findings clarify that successful AI adoption operates across multiple levels—including documentation infrastructure, workflow uptake, and individual user acceptance—highlighting why model accuracy alone is insufficient to ensure real-world impact. These results provide real-world evidence that methodologically grounded, structurally informed LLM systems can achieve robust, equitable, and operationally meaningful performance in clinical documentation workflows.

## Introduction

The International Classification of Diseases (ICD) is foundational to hospital billing, epidemiologic surveillance, and health-system operations. National adaptations, such as ICD-10-CM for diagnoses and ICD-10-PCS for procedures in the United States, reflect local clinical and administrative needs. Manual ICD-10 coding, however, remains labor-intensive and prone to errors. In Taiwan, certified coding specialists (CCSs) require an average of 22 min per inpatient record, and U.S. coders report approximately 69 percent longer times following the transition from ICD-9‑CM to ICD-10-CM^1^. As documentation volume and case complexity continue to rise, the scalability of manual coding has become a persistent operational challenge for hospitals.

As artificial intelligence (AI) transitions from controlled research settings into hospital workflows, successful workflow-level adoption depends not only on predictive accuracy but also on workflow fit—how well the tool integrates into existing processes—and on coder-level acceptance, including trust, perceived usefulness, and accountability. Within this context, automated coding provides a practical testbed for exploring these dynamics and examining the feasibility and human factors of AI-assisted decision support. Early machine learning approaches have largely been replaced by deep learning architectures, such as hierarchical attention networks (HAN)^2^ and bidirectional gated recurrent units (BiGRU)^3^, trained on benchmark datasets such as MIMIC-III^4^ to exploit clinical text representations for ICD coding prediction. However, most of these datasets remain limited to ICD-9 codes and lack consistent infrastructure-level documentation standards, constraining their utility for real-world ICD-10 modeling where both terminological complexity and clinical documentation variability are high.

Large language models (LLMs) offer new opportunities for medical code generation^5–7^. Instruction-tuned decoder models, such as GPT-2, have demonstrated improved flexibility compared to encoder-only counterparts and have been applied to ICD coding tasks with moderate success^8,9^. However, despite domain-specific pretraining and the use of ontology-enriched prompts^10^, direct prompting with general-purpose LLMs still suffers from hallucination of invalid codes, insufficient grounding in ICD taxonomies^11–13^, and poor alignment with clinical context-limitations that hinder deployment in routine clinical workflows.

In parallel, the increasing adoption of Health Level Seven (HL7) Clinical Document Architecture (CDA)^14^ and related documentation standards has facilitated the segmentation of medical records into consistent and interpretable sections across healthcare systems^15^. Structured sections such as discharge diagnosis (DischgDiag), medical history (MedHist), and operation note (OpNote) provide stable semantic anchors for diagnostic reasoning and represent a largely underexplored resource for clinical AI development. Despite this opportunity, few studies have systematically evaluated how HL7-aligned section content affects LLM performance in real-world ICD-10-CM coding. Rather than treating HL7 CDA as an intervention, our study leverages its existing institutional adoption as a methodological precondition for section-aware modeling and cross-institutional evaluation. Furthermore, clinical documents are often highly redundant, with frequently assigned codes recurring in near-duplicate narrative contexts^16^ leading to the risk of overfitting if not properly managed during model training.

Recent work underscores that the effectiveness of LLMs in clinical coding hinges on multiple factors-not only model architecture and careful curation of training data, but critically, the selection of an appropriate base model for fine-tuning. The latter substantially shapes downstream performance^17^ by determining how well the model captures the intrinsic understanding of ICD code definitions^12^. As the number of pretrained models continues to expand, exhaustive fine-tuning across all candidates has become increasingly impractical, particularly in hospital information technology (IT) environments^18^, highlighting the need for scalable and principled model selection strategies prior to downstream fine-tuning.

Finally, despite rapid LLM progress in task-specific fine-tuning^6,8,9,19^, benchmark gains do not always translate into real-world clinical impact. The evaluations of most studies^20,21^ rely on synthetic or publicly available datasets that lack the structural richness and contextual complexity of real hospital documentation. As a result, few investigations have engaged CCSs in validating model behavior within operational hospital workflows, leaving the generalizability, deployment readiness, and user acceptance of developed models underexplored^22^. Understanding these human-AI dynamics is crucial for the responsible deployment and sustainable integration of AI into clinical workflows.

To bridge this translational gap, we developed an integrated and modular framework for automated ICD-10-CM coding combining principled base model selection, HL7-aligned section-aware prompting, and redundancy-aware training. We further conducted a 13-week, human-in-the-loop pilot randomized controlled trial (RCT) involving ten CCSs at a medical center in Taiwan, embedding three AI-assisted models (PubMedGPT-2, HAN, and BioMistral^23^) directly into routine coding operations. This pilot study allowed us to jointly evaluate algorithmic performance, coding efficiency, and user-centered outcomes.

Specifically, we addressed two research questions (RQs):

RQ1. What impact do AI-assisted workflows have on the time required to complete ICD-10-CM coding tasks in real clinical practice?

RQ2. How do model type and coder characteristics (demographic and professional) influence coder-level acceptance of AI and satisfaction within the real-world ICD-10-CM workflow?

## Results

### Modular pipeline for developing an ICD-10-CM coding system

**Fig. 1:**
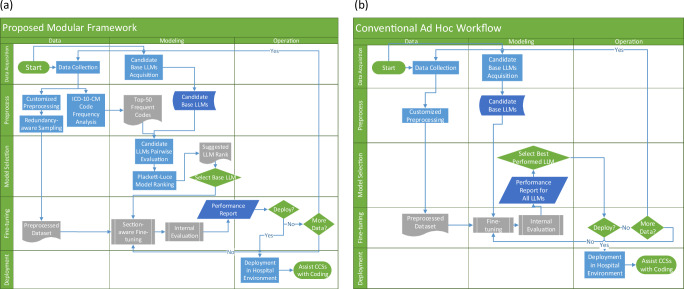
Overview of the proposed modular framework for developing an International Classification of Diseases, Tenth Revision, Clinical Modification coding system. **a** The panel depicts a structured workflow comprising data acquisition, redundancy-aware sampling, large language model (LLM)-as-judge evaluation with Plackett-Luce ranking, section-aware fine-tuning, and clinical deployment. **b** The panel contrasts this with conventional, ad hoc pipelines that lack principled model selection and incur significant inefficiencies during development. This figure illustrates the algorithmic development pipeline and does not depict the randomized controlled trial design, which is described separately in the Methods section.

Figure 1a presents the modular pipeline developed for ICD-10-CM coding, emphasizing a principled, data-driven alternative to conventional intuition-based workflows. The pipeline begins with data acquisition, which includes identifying the top 50 most frequently occurring ICD-10-CM codes and collecting candidate LLMs. A redundancy-aware sampling strategy is then applied to reduce semantically redundant discharge summaries. Candidate LLMs are first screened using pairwise LLM-as-judge^24^ comparisons to assess their intrinsic semantic alignment with ICD-10 code definitions. Rather than serving as a definitive measure of coding performance, this intrinsic evaluation functions as a pragmatic, low-cost heuristic for narrowing the set of candidate foundation models under deployment constraints. Partial or complete comparison outcomes are aggregated using a Plackett-Luce model^25,26^ to derive a global preference ranking without requiring exhaustive fine-tuning of all candidates.

The suggested base model is subsequently fine-tuned using section-aware instruction prompts derived from HL7-CDA R2-aligned discharge summaries, enabling adaptation to heterogeneous document structures encountered in real-world clinical practices. The resulting model is then deployed within the hospital’s coding workflow to assist CCSs during routine ICD-10-CM assignment. In parallel, we prospectively recorded coding curation time and user-reported satisfaction in an operational clinical environment, enabling real-world evaluation of the pipeline components and providing empirical grounding for answering our two RQs based on the hypotheses outlined in the Method section.

The modular pipeline stands in contrast to conventional pipelines shown in the Fig. 1b, which rely on ad hoc model selection and exhaustive fine-tuning of all candidates, leading to inefficient resource use and limited scalability. The following sections detail the results of each component in this pipeline, from data collection to human-in-the-loop deployment.

### Data collection results

We assembled three anonymized, HL7-CDAR2-compliant datasets from two hospitals in Taiwan to support model development, external validation, and real-world evaluation. The primary dataset, derived from Kaohsiung Medical University Chung-Ho Memorial Hospital (KMUH) in Kaohsiung, consisted of 125,820 discharge summaries collected between April 2019 and March 2021 after data cleaning (see Supplementary Note 1). These records contained 11,991 unique ICD-10-CM codes and were stratified into training (100,656), validation (12,582), and internal test sets (12,582). Chapter-level ICD-10-CM distributions across these splits are shown in Supplementary Fig. 1.

In consultation with CCSs at KMUH, the DischgDiag section was identified as the primary anchor for ICD coding, with MedHist, OpNote, pathology report (PathRep), and treatment course (TreatCous) referenced most frequently from the remaining 13 observed section headers (see Supplementary Table 1). All records included the DischgDiag section. MedHist is the most frequently co-occurring section, followed by OpNote, PathRep, and TreatCous. Over half of the records contained all five sections, reflecting the widespread adoption of HL7-aligned documentation at the infrastructure level in KMUH. Approximately 85% of the training samples fall within the 2048-token limit used for downstream experiments due to hardware constraints; longer samples were truncated using a priority-based strategy described in the Methods section. Further explanations of section combinations and token statistics are provided in Supplementary Note 2 and Supplementary Table 2, respectively.

**Fig. 2:**
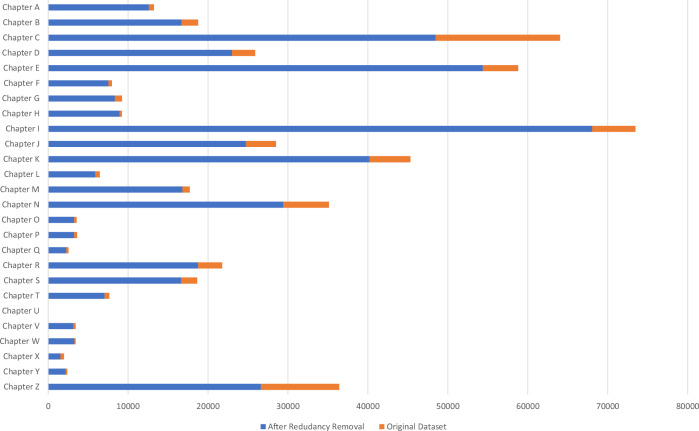
ICD-10-CM (International Classification of Diseases, Tenth Revision, Clinical Modification) chapter distribution (A-Z) in the training set before and after redundancy-aware sampling. This figure compares the distribution of ICD-10-CM codes by chapters in the Kaohsiung Medical University Chung-Ho Memorial Hospital training set before and after applying the redundancy removal procedure.

Figure 2 visualizes the ICD-10-CM chapter-level distribution of codes before and after applying redundancy-aware sampling. All chapters experienced reductions in sample counts, with the largest proportional decrease observed in Chapter C (Neoplasms, ~14.7%).

To assess real-world applicability and generalizability, two external datasets were collected. The first consisted of retrospective summaries from Taitung MacKay Memorial Hospital (TMMH; from January 2021 to December 2022), providing a cross-institutional test set without retraining. The HL7-CDA-aligned documentation infrastructure at both institutions enabled a systematic comparison of section-level composition across datasets. As summarized in Supplementary Table 3, all TMMH discharge summaries contained the DischgDiag section, but section co-occurrence patterns differed markedly from those observed at KMUH. In contrast to KMUH, where over half of the records contained all five commonly used sections (DischgDiag, MedHist, OpNote, PathRep, and TreatCous; Supplementary Table 2), TMMH records exhibited a more restricted and heterogeneous structure. The most prevalent configuration at TMMH consisted of DischgDiag + MedHist + TreatCous, while combinations involving OpNote and PathRep were substantially less frequent. Furthermore, all TMMH summaries fell well below the 2048-token limit used in downstream experiments, and no truncation was applied to the TMMH dataset. These characteristics position TMMH as a meaningful stress test for evaluating model robustness under distinct real-world documentation practices without retraining.

The second external set was curated prospectively during the KMUH RCT conducted between October and December 2024, in which 10 CCSs evaluated three AI-assisted workflows (HAN, PubMedGPT-2, and BioMistral) alongside manual coding following the deployment procedure presented in our previous work^8^ at KMUH. Of 12,455 collected summaries, 10,688 records passed quality filtering (Supplementary Note 1) and were used to characterize post-deployment model performance and coding efficiency in relation to the two RQs. Further details on the RCT design and implementation are provided in the Methods section. Summary statistics for both external datasets are shown in Supplementary Fig. 2.

### Model selection results for the ICD-10-CM coding task

To identify an optimal base model for downstream ICD-10-CM coding, we conducted an intrinsic screening evaluation of five decoder-only LLMs under 7 billion (B) parameters: PubMedGPT-2^8^, Llama2-7B^27^, Mistral-7B instruct^28^, and their domain-adapted variants MedLlama2 and BioMistral-7B^23^. Each model was assessed using pairwise LLM-as-judge comparisons on single-code definitions of the 50 most frequent ICD-10-CM codes in the training corpus. Representative examples and judgment results are provided in Supplementary Note 3.

**Fig. 3:**
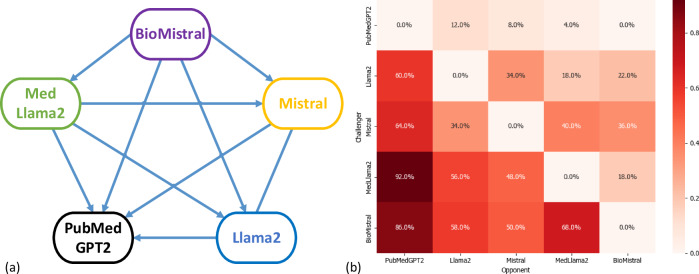
Intrinsic model selection via pairwise Large Language Model (LLM)-as-judge evaluation. **a** The panel visualizes the win-loss relationships among models using a mixed directed graph, where each node denotes a candidate base model. A directed edge from model A to model B indicates that A was preferred over B by the judge in the ICD-10-CM (International Classification of Diseases, Tenth Revision, Clinical Modification) code definition generation task; undirected edges indicate ties when no clear preference was expressed. The outdegree of each node reflects the number of wins achieved by the corresponding model. **b** The panel shows a win-rate matrix heatmap, where each cell reports the proportion of comparisons in which a challenger model (row) was preferred over a specific opponent model (column) in generating definitions for the top 50 most frequent ICD-10-CM codes. These values represent local, opponent-specific outcomes rather than overall model performance. Global model preference is obtained by aggregating all pairwise outcomes using the Plackett–Luce model.

As shown in Fig. 3, BioMistral achieved the highest overall win rates across pairwise matchups, followed by MedLlama2 and Mistral. Maximum-likelihood Plackett–Luce aggregation (see Methods) yielded selection probabilities of 44.1% for BioMistral, 22.6% for MedLlama2, and 19.2% for Mistral, identifying BioMistral as the most suitable base model for fine-tuning. PubMedGPT-2 and Llama2 exhibited markedly lower intrinsic alignment with ICD-10-CM definition (1.7% and 12.4%, respectively). Robustness checks using incomplete comparison matrices showed consistent model ordering (Supplementary Note 4).

These findings provide an intrinsic, definition-level ranking of candidate architectures and serve as a pragmatic, low-cost heuristic to prioritize candidate base models for downstream fine-tuning, rather than a definitive measure of coding performance. Accordingly, we selected BioMistral for all downstream fine-tuning experiments.

### ICD-10 coding task performance and redundancy-aware sampling improvements

**Fig. 4:**
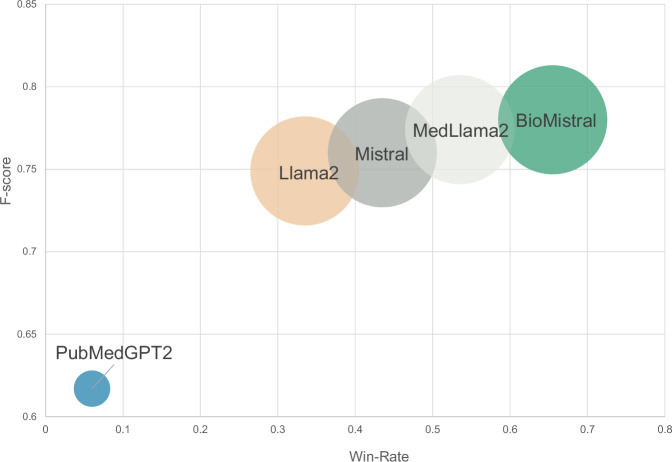
Fine-tuned model performance across average win-rate (x-axis), F_1_-scores (y-axis), and parameter size (bubble size). The figure illustrates a comparative analysis of the F-scores of five developed decoder-based models in relation to the intrinsic rankings inferred by our algorithm. Models toward the upper-right corner reflect stronger performance and greater consistency with intrinsic preference ordering, rather than definitive superiority.

To examine the downstream consistency of the intrinsic model rankings derived from the LLM-as-judge evaluation, all five candidate base models were fine-tuned using only the DischgDiag section and evaluated on the internal test set. As shown in Fig. 4, the relative ordering of models was preserved after fine-tuning: BioMistral achieved the highest downstream performance (F_1_-score = 0.78; win rate = 69.2%), followed by MedLlama2 and Mistral. Despite sharing a similar parameter size of 7B, Llama2 underperformed relative to its counterparts, whereas PubMedGPT-2, despite being pretrained on biomedical abstracts, showed the lowest F_1_-score. These results indicate a consistent association between intrinsic ICD-10-CM definition-level preference rankings and downstream coding performance within the evaluated model set. Importantly, we do not interpret this alignment as evidence of a causal relationship, but rather as supportive empirical evidence that the intrinsic screening task can serve as a pragmatic heuristic for narrowing candidate models prior to fine-tuning. This observation supports the practical utility of the proposed principled model selection method when combined with downstream validation, particularly under real-world constraints where exhaustive fine-tuning of all candidate foundation models is infeasible.

Table 1 presents a comparative evaluation of nine models spanning deep learning-based, encoder-based, and decoder-based architectures, all trained and evaluated under identical dataset splits and experimental settings, rather than being directly adopted from prior literature. Decoder-based models consistently outperformed other approaches on both full-code and top-50-code prediction tasks. While some non-decoder models achieved higher precision, particularly BiGRU and BERT, all showed substantially lower recall, limiting their utility for comprehensive ICD-10-CM coding tasks. Notably, the weaker recall of encoder-based transformers in Table 1 is consistent with prior evidence that BERT-style encoders do not automatically translate into strong ICD assignment performance, especially under long-document and full-code settings^6,29^. Furthermore, PLM-ICD—a strengthened BERT variant—showed comparatively more stable behavior than vanilla BERT, yet still underperformed decoder-based LLMs, which better sustained recall under real-world ICD-10-CM complexity and cross-institution variability. BioMistral delivered the best overall performance across both tasks, supporting that decoder-based LLMs, within the evaluated model families under our real-world setting, and when guided by principled model selection and fine-tuning, demonstrated more reliable performance than encoder-based and traditional deep learning baselines for ICD-10-CM coding.

**Table 1:** Performance comparison across architectures on the real-world ICD-10-CM coding task

Architecture	Model	Full codes			Top-50 codes		
		P	R	F	P	R	F
Deep Learning-based	BiGRU	0.799	0.445	0.571	0.855	0.741	0.794
	HAN	0.518	0.535	0.526	0.857	0.773	0.812
Encoder-based	BERT	**0.889**	0.316	0.466	0.894	0.730	0.804
	PLM-ICD	0.763	0.610	0.678	0.854	0.830	0.842
Decoder-based	PubMedGPT-2	0.655	0.671	0.663	0.854	0.866	0.860
	Llama2	0.756	0.741	0.749	0.861	0.875	0.868
	MedLlama2	0.786	0.762	0.774	0.880	0.880	0.880
	Mistral-instruct	0.764	0.756	0.760	0.873	**0.881**	0.877
	BioMistral	0.795	**0.765**	**0.780**	**0.917**	0.880	**0.906**

Bolded scores represent the best performance in each column. Decoder-based models outperform other baselines in both full-code and top-50-code settings.

**Fig. 5:**
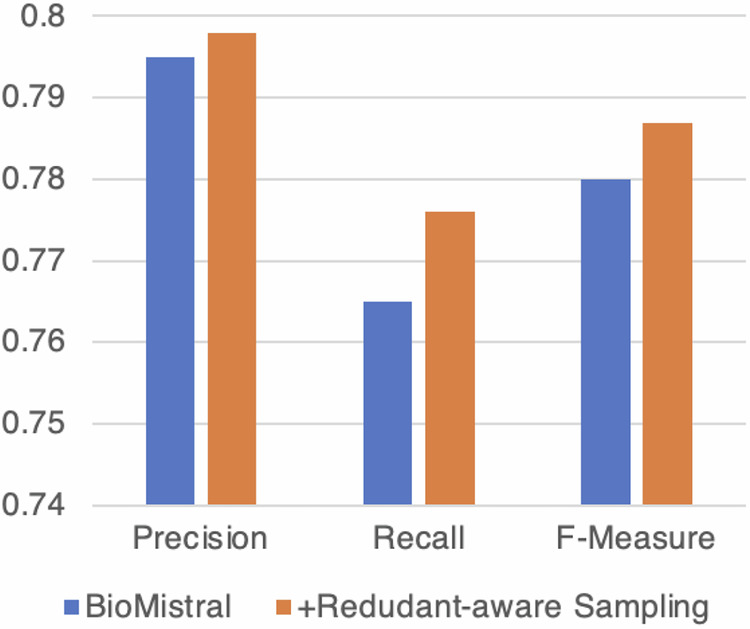
Performance comparison of BioMistral models trained on full versus deduplicated datasets. Results are reported on the full test set with 12,582 test samples.

To evaluate the effectiveness of the redundancy-aware sampling strategy, we further compared BioMistral models trained on the full dataset using only the DischgDiag section as input versus a deduplicated dataset constructed via redundancy-aware sampling. As shown in Fig. 5, the deduplicated model consistently outperformed the baseline across precision, recall, and F_1_-scores, and reduced total training time by 10.2%. These findings reinforce prior work in clinical natural language processing^30–32^, which has shown that removing semantically redundant samples not only accelerates training and mitigates overfitting, but also enhances model generalization-particularly important when adapting to diverse documentation styles or deploying across institutions. These dual improvements further highlight the utility of removing semantically redundant summaries-particularly in resource-constrained hospital IT environments—where minimizing training costs and mitigating overfitting are operationally important.

### Impact of section-wise content inclusion on model performance

To investigate how structured section-wise content influences ICD-10-CM coding performance, we compared a universal BioMistral model—trained to handle heterogeneous HL7-CDA Release 2 (CDAR2) section combinations using a unified prompt format—with five section-combination-specific BioMistral models trained on fixed and distinct combinations of HL7-CDAR2 sections and evaluated only on a fixed input structure. Two evaluation settings were used: (1) a full test set containing all test samples regardless of their section composition, and (2) matched test subsets restricted to test samples whose section combinations aligned with each section-combination-specific model during the training phase. This comparison leverages existing documentation infrastructure rather than introducing new documentation practices, allowing us to isolate modeling strategies under real-world constraints. The evaluation protocol is detailed in Supplementary Note 5.

**Fig. 6:**
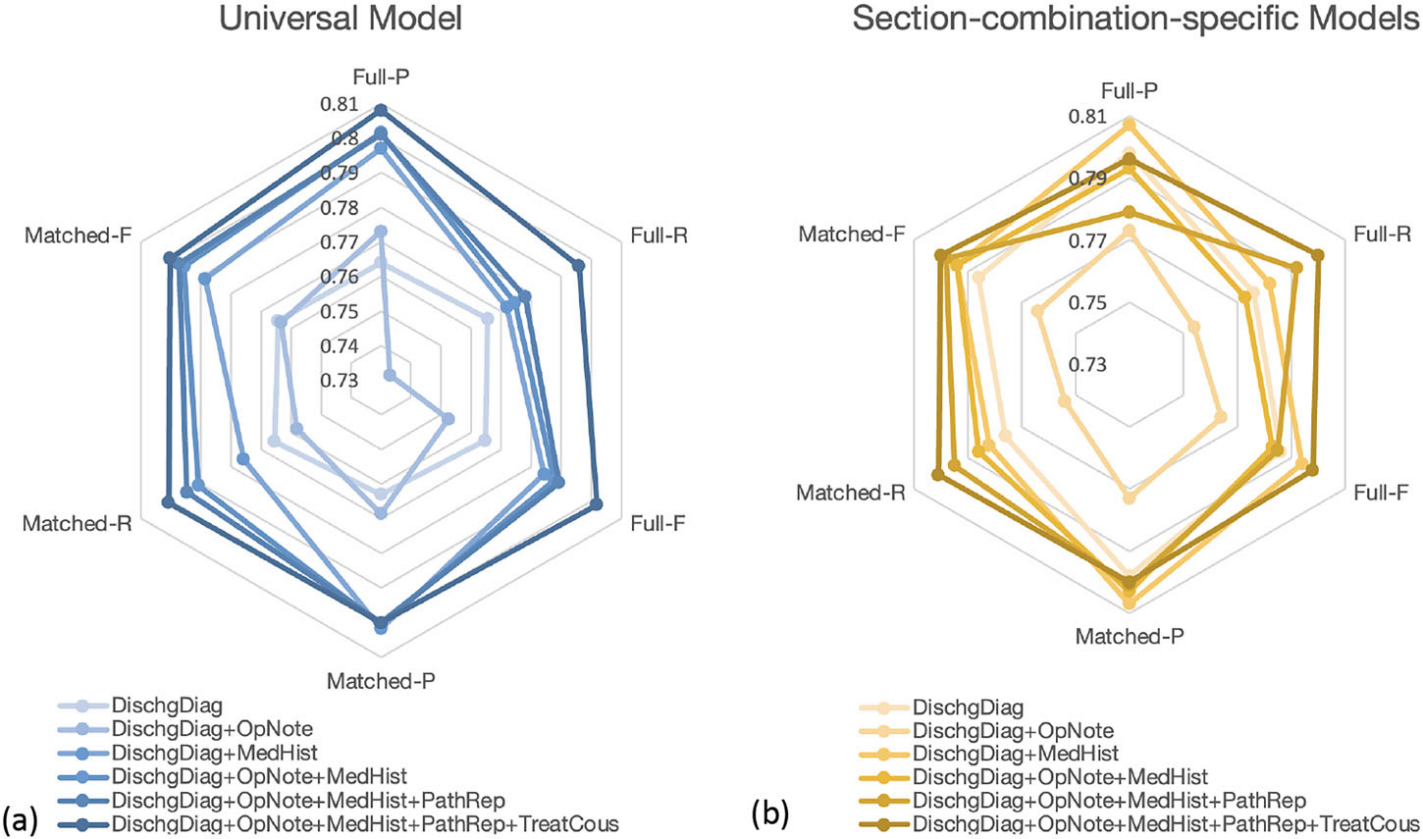
International Classification of Diseases, Tenth Revision, Clinical Modification coding performance comparison for Health Level Seven Clinical Document Architecture Release 2 section-wise content inclusion during model training. **a** The panel shows results for a single universal model trained across all available section combinations using a unified prompt format. **b** The panel displays results for five section-combination-specific models, each trained on a distinct combination of clinical sections (e.g., Discharge Diagnosis (DischgDiag) section only, DischgDiag + Operative Note section, etc.). Performance is evaluated on both the full test set and the matched test subset corresponding to each section configuration.

As shown in Fig. 6 and Supplementary Table 5, incorporating additional HL7-CDAR2 sections generally improved F-scores across all models on the matched subsets, suggesting that richer structured inputs help models better capture diagnostic cues. MedHist contributed the most substantial gains; the DischgDiag+MedHist configuration achieved an F-score comparable to that of the full-section model, underscoring that historical clinical information provides a strong diagnostic signal. However, according to ICD-10-CM coding guidelines, not all diagnoses documented in MedHist are eligible for coding; only comorbidities and prior conditions that actively influence management, treatment, or evaluation during the current admission should be assigned codes. In practice, MedHist often contains a mixture of active and inactive conditions, and a naive reliance on this section alone may increase recall at the cost of precision, thereby raising the risk of over-coding in reimbursement-sensitive settings.

In contrast, OpNote alone produced minimal benefit, consistent with its limited use for broad diagnostic coverage outside procedure-specific contexts. Nevertheless, OpNote frequently serves as the most authoritative source of definitive diagnosis in surgical admissions, where intraoperative findings or pathology-confirmed results establish the final ICD-10-CM diagnosis. This is reflected in our results: when paired with MedHist, OpNote modestly but consistently improved precision, particularly in the universal model, suggesting a complementary role in confirming and refining diagnostic assignments rather than expanding diagnostic hypotheses. Moreover, many ICD-10-CM codes rely on fine-grained anatomical specificity, details that are often documented only in OpNote and are critical for diagnosis-related groups assignment and reimbursement weighting.

On the full test, the universal model exhibited consistent, monotonic improvements in both precision and recall as more sections were included in the input, whereas section-specific models tended to perform optimally only when the input structure exactly matched their training configuration. This contrast highlights a key trade-off: while section-specific training can yield strong performance under tightly controlled and structurally aligned conditions, it does not generalize reliably to heterogeneous real-world documentation. From an exploratory perspective, the section-specific models therefore represent an informative but ultimately limited attempt to improve performance through structural specialization. In contrast, the universal model provides superior robustness to missing or inconsistently populated sections—an empirical property that is essential for scalable deployment in routine clinical environments, where strict section completeness cannot be assumed.

Finally, comparing these results from redundancy-aware sampling revealed complementary effects on downstream performance: the F-score increased from 0.780 (baseline) to 0.786 ( + deduplication) and 0.798 ( + full section inclusion). Together, these findings show that semantic deduplication and structured content enrichment jointly enhance the reliability of ICD-10-CM coding under real-world heterogeneous documentation conditions.

### Robustness and generalizability of ICD-10-CM coding models

Using the same set of models as Table 1, Table 2 summarizes external validation results on two independent datasets: (1) the KMUH RCT dataset collected during live clinical deployment, and (2) the retrospective dataset from TMMH, which reflects a distinct documentation style and section completeness profile. This evaluation assesses real-world operational performance and cross-institutional generalizability without additional retraining.

**Table 2:** ICD-10-CM coding task performance on external datasets

Dataset	Model	Full codes			Top-50 codes		
		P	R	F	P	R	F
KMUH RCT	BiGRU	0.780	0.369	0.501	0.852	0.683	0.758
	HAN	0.335	0.166	0.222	0.448	0.271	0.338
	BERT	**0.875**	0.278	0.421	0.883	0.673	0.764
	PLM-ICD	0.763	0.654	0.678	0.854	0.832	0.842
	PubMedGPT-2	0.702	0.423	0.528	0.784	0.551	0.647
	Llama2	0.754	0.694	0.723	0.873	0.858	0.866
	MedLlama2	0.762	0.714	0.737	0.884	0.865	0.875
	Mistral	0.779	0.696	0.736	0.892	0.820	0.855
	BioMistral	0.784	0.727	0.754	0.890	0.867	0.878
	+Redundancy Removal	0.794	0.737	0.764	0.901	0.888	0.894
	+All sections	0.807	**0.752**	**0.779**	**0.929**	**0.910**	**0.920**
TMMH	BiGRU	0.221	0.158	0.184	0.310	0.223	0.260
	HAN	0.300	0.147	0.197	0.434	0.251	0.318
	BERT	0.137	0.100	0.115	0.809	0.473	0.596
	PLM-ICD	0.598	0.550	0.573	0.743	0.682	0.711
	PubMedGPT-2	0.374	0.339	0.356	0.481	0.439	0.459
	Llama2	0.657	0.562	0.605	0.789	0.681	0.731
	MedLlama2	0.659	0.579	0.616	0.794	0.690	0.739
	Mistral	0.671	0.573	0.618	0.786	0.674	0.728
	BioMistral	0.667	0.579	0.620	0.834	0.675	0.746
	+Redundancy Removal	0.673	0.585	0.626	**0.845**	0.681	0.754
	+All sections	**0.685**	**0.594**	**0.636**	0.832	**0.695**	**0.757**

The upper part of the table corresponds to KMUH test data collected during routine ICD-10-CM workflows post-deployment. The lower part reports results on the retrospective dataset from TMMH. Bolded values indicate the best performance per metric.

Across both external datasets, decoder-based LLMs consistently outperformed traditional deep learning and encoder-based baselines. At KMUH, performance patterns closely mirrored those observed on the internal test set (Table 1). Specifically, BioMistral achieved the highest F-score among all models (0.754 for full codes; 0.878 for top-50 codes), followed by MedLlama2 and Mistral. In contrast, non-decoder-based models exhibited substantially low recall, limiting their utility in practically comprehensive diagnostic coding tasks.

Performance declined for all models on the TMMH dataset, reflecting the expected impact of institution-specific documentation variability, including differences in documentation conventions and section availability. As shown in Supplementary Fig. 2, the dominant ICD-10-CM chapter distributions differ substantially between institutions, and Supplementary Table 3 further indicates greater heterogeneity in section completeness at TMMH. The drop was most pronounced among non-decoder architectures (e.g., BERT precision dropped from 0.875 at the source hospital to 0.137 at TMMH). Decoder-based LLMs with 7B parameters demonstrated markedly stronger robustness under these institutional and documentation-level distribution differences, maintaining F-scores in the range of 0.60-0.75. BioMistral again ranked among the top performers (0.620 full-code F-score), with performance further improved by redundancy-aware sampling ( + 0.006) and by incorporating all HL7-CDAR2-compliant sections ( + 0.016).

Taken together, these findings show that decoder-based LLMs exhibit greater stability across heterogeneous institutional documentation environments, rather than across different task domains. Improvements observed from redundancy-aware sampling and structured section inclusion further support the modular pipeline’s design as a generalizable and deployment-oriented approach to ICD-10-CM coding across real-world hospital deployments.

### Human-in-the-loop pilot results from KMUH RCT

A total of 11 CCSs were enrolled in the 13-week pilot RCT conducted at KMUH between October and December 2024. One late-career CCS closing retirement withdrew early due to unwillingness to adopt the AI-assisted interface (Supplementary Fig. 3) and refused to complete the 5-point Likert scale survey as shown in Supplementary Fig. 4, was excluded from all analyses, leaving an analytic sample of 10 CCSs (mean experience: 12 years; spanning Generation (Gen) X, Y, and Z^33,34^). Demographic and professional background characteristics are provided in Supplementary Note 6 and Supplementary Table 6.

The implementation of the AI-assisted ICD-10-CM coding workflow was supported by departmental leadership, coordination with the hospital’s information technology team, and targeted user training sessions designed to facilitate smooth integration into existing coding practices. Notably, CCSs did not encounter a new interface at the start of the RCT: the coding interface had been available for pilot use since February 2023 in a manual-entry mode and was iteratively refined based on CCS feedback prior to the introduction of AI assistance in October 2024. Following these preparatory efforts, workflow-level uptake of the AI-assisted workflow increased steadily from 37.26% in October to 79.63% in November and 90.59% in December 2024, indicating rapid operational uptake among CCSs. Workflow-level uptake was defined as the proportion of discharge summaries coded with AI assistance among all summaries coded by the enrolled CCSs within each time period.

**Fig. 7:**
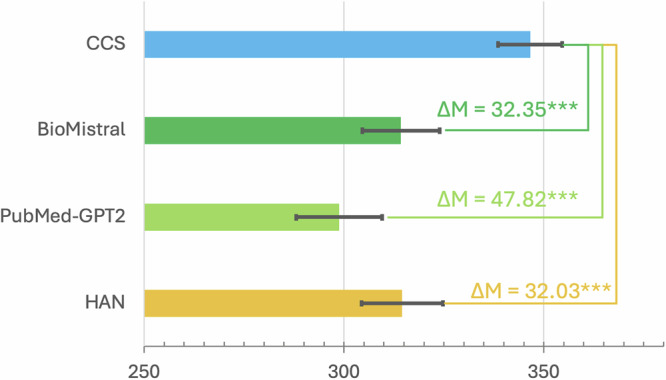
Differences in average coding time (seconds) across artificial intelligence (AI)-assisted and manual coding workflows. Bar plots show the mean coding time (in seconds) required per case under manual coding versus three AI-assisted workflows: hierarchical attention network (HAN), PubMedGPT-2, and BioMistral. Time savings (ΔM) are calculated relative to the manual baseline. All AI-assisted workflows were associated with significantly reduced task durations (^***^P < 0.001, Games-Howell). Workflow assignment was randomized on a weekly rotation basis across certified coding specialists (CCSs) during a 13-week trial. CCSs were aware of whether AI assistance was present but were blinded to the identity of the specific AI model used in each AI-assisted condition. Error bars represent 95% confidence intervals.

To evaluate RQ1, we first analyzed the coding time per summary across all sessions conducted with or without AI support. Across 10,688 coded summaries, significant differences in mean coding time were observed between manual and AI-assisted workflows (Welch’s ANOVA, *p* < 0.001). All three AI-assisted workflows yielded faster completion times than manual coding (Games-Howell, all *p* < 0.001). As illustrated in Fig. 7, PubMedGPT-2 produced the largest average time reduction, followed by BioMistral and HAN. These findings support the hypothesis underlying RQ1 and demonstrate that AI assistance can materially reduce CCS workload under real-world conditions.

**Fig. 8:**
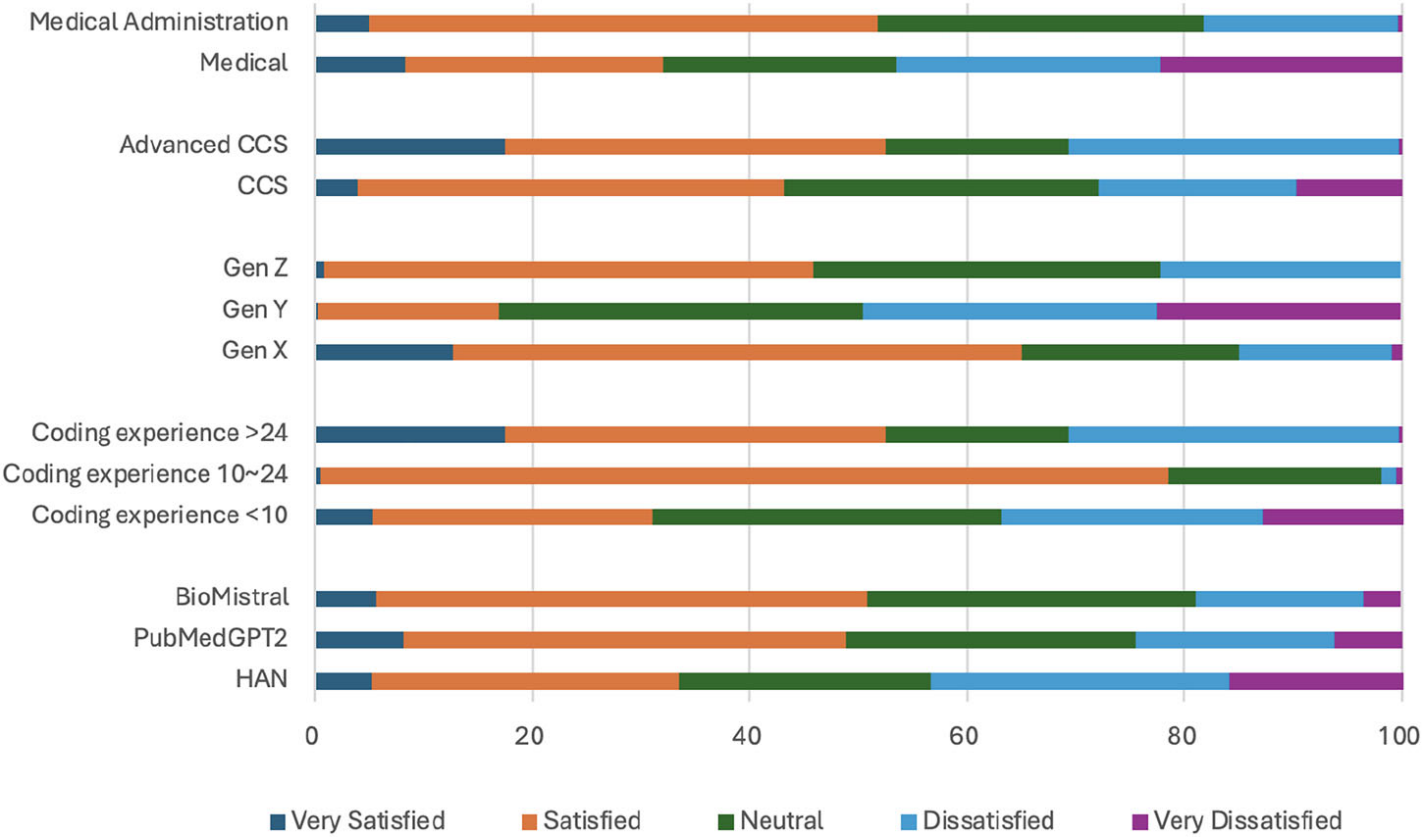
Satisfaction levels of certified coding specialists (CCSs) stratified by model type and background characteristics. The figure presents the percentage distribution of satisfaction levels (Very Satisfied, Satisfied, Neutral, Dissatisfied, Very Dissatisfied) across key CCS subgroups, including education background, certification level, years of coding experience, generation cohort, and artificial intelligence (AI) model exposure. Survey responses were collected anonymously during live AI-assisted International Classification of Diseases, Tenth Revision, Clinical Modification coding tasks throughout a 13-week randomized controlled trial. The figure reveals notable variation in satisfaction patterns, highlighting subgroup-specific trends in model usability and perceived value.

To further understand CCS perceptions (RQ2) and hypotheses H2a and H2b defined in the Methods section, we analyzed a total of 1947 valid satisfaction responses collected during the RCT period. As illustrated in Fig. 8, satisfaction varied across both model type and coder background characteristics. Among the three models, BioMistral received the highest proportion of positive ratings, followed by PubMedGPT-2 and HAN, consistent with their relative technical performance. Satisfaction also varied by CCS background: higher satisfaction was reported among CCSs with healthcare administration training, advanced certification, 10–24 years of experience, and members of Gen X, whereas lower satisfaction was more common among CCS with medical-related academic backgrounds, those with <10 years of experience, and members of the Gen Y cohort (*χ*², *p* < 0.001).

To evaluate hypothesis H2a, we conducted a multinomial logistic regression using BioMistral and the “Very Dissatisfied” response as joint reference categories. Despite all AI-assisted workflows achieving significant time savings, clear differences in satisfaction were observed across models. Although HAN provides an explainable interface through highlighting predicted ICD-10-CM key terms^8^ and was associated with the reduction of the coding time, users of the HAN model were less likely to provide positive or neutral ratings, whereas PubMedGPT-2 showed significantly reduced odds of satisfaction across “Neutral” and “Satisfied”, but no significant difference was observed for “Very Satisfied”. The finding that BioMistral consistently received more favorable satisfaction scores aligns with the relative technical performance profiles of the three models and prior evidence that domain-specific models enhance perceived usefulness and trust in AI applications^35^. Supplementary Table 7 and Supplementary Note 7 provide further details on the regression estimates and subgroup variability.

To explore H2b, we examined coder background characteristics using separate multinomial logistic regressions for each subgroup (Supplementary Tables 8–11). Several patterns emerged. CCSs having <10 years of experience showed significantly lower odds of selecting higher satisfaction categories compared with those with over 25 years of experience, whereas mid-career coders (10–25 years) exhibited mixed effects across satisfaction levels. Certification level was also associated with satisfaction: coders holding advanced CCS certification reported higher satisfaction probabilities than those with standard certification. Educational background further influenced satisfaction, with clinically trained CCSs being less likely to provide favorable ratings compared to those with healthcare administration training. After collapsing sparse categories, we also observed generational differences: with Gen Y as the reference group, both Gen X and Gen Z showed significantly higher odds of reporting “Satisfied” or “Neutral” responses, indicating that Gen Y consistently expressed the lowest satisfaction. This U-shaped pattern across age cohorts may reflect distinct attitudes toward trust, digital familiarity, or cognitive workload expectations when interacting with AI.

Because only ten CCS contributed repeated measures in this pilot RCT, these regression results should be interpreted cautiously and viewed as exploratory signals rather than definitive statistical evidence. The complete set of estimates, subgroup trends, and robustness checks is provided in Supplementary Note 7.

## Discussion

Our findings suggest that domain-specialized, decoder-based LLMs—particularly BioMistral—offer the most consistent and generalizable performance for ICD-10-CM coding in real-world clinical settings within the scope of the evaluated model families. Across internal validation and external evaluation on independent hospital datasets, the model identified through our principled base model selection framework consistently ranked among the top-performing approaches. Importantly, performance remained stable despite substantial variation in documentation style and institutional conventions, underscoring practical robustness to institutional and documentation-level variability—a central requirement for AI systems deployed in dynamic and heterogeneous clinical environments. Rather than asserting universal superiority, these results highlight the value of systematic, evidence-informed model selection in clinical AI pipelines, particularly in light of the rapid proliferation of foundation models.

Our selection framework introduces several methodological advances by combining pairwise LLM-as-judge comparisons with Plackett–Luce aggregation. This design enables fine-grained discrimination among candidate architectures without requiring exhaustive pairwise evaluations and fine-tuning of all candidates, a practical advantage as the landscape of foundation models continues to evolve. Our intrinsic single-code definition task (see Methods) serves as a focused probe of definition-level ICD-10-CM knowledge, yielding preference rankings that diverged from those reported by Lee and Lindsey^12^, whose multi-code generation paradigm introduces confounding effects related to instruction-following and output formatting. We emphasize that this intrinsic task is intended as a screening heuristic rather than a definitive predictor of downstream coding performance, and its utility should be interpreted in conjunction with subsequent fine-tuning and external validation.

Importantly, we explicitly designed the evaluation framework to mitigate potential bias and instability inherent in automatic LLM-based judging. First, global model preference is inferred through Plackett-Luce aggregation, which integrates evidence from multiple noisy pairwise comparisons rather than relying on a single judgment. Second, the framework allows ties when the judge model does not express a clear preference, avoiding forced or spurious distinctions between closely matched candidates^24^. Third, robustness analyses shown in Supplementary Note 4 further show that our aggregation yields stable rankings even when comparison matrices are incomplete, underscoring the practicality of this framework for real-world model selection where exhaustive comparisons may be infeasible. Together, these design choices emphasize relative and aggregated preference signals rather than absolute judgments, enabling a transparent, scalable, and reproducible base-model selection. By design, our framework prioritizes practical decision support for model narrowing over theoretical guarantees of optimality.

Beyond architectural choices, cross-institutional robustness reflects the interaction of modeling strategies with real-world documentation ecosystems. Three factors likely contributed to the developed model’s stability across hospitals. First, redundancy-aware sampling reduced overrepresentation of duplicated or structurally homogeneous summaries, broadening exposure to heterogeneous narrative patterns. Second, universal prompting strategies demonstrated greater resilience to missing or inconsistently used sections compared with section-specific models, which, while accurate on format-aligned subsets, were more sensitive to structural variations. Finally, distinct from model-level considerations, the shared documentation infrastructure-level adoption of HL7-CDAR2–based structured records across participating hospitals provided recurring section templates and partially harmonized information architectures. This partial standardization—rarely emphasized in prior ICD coding studies—likely contributed to smoother cross-institutional generalization. Collectively, these findings suggest that robustness in clinical LLMs emerges not solely from model design, but also from the alignment between model training strategies, documentation infrastructure, and deployment context.

Notably, our study operationalizes “adoption” as a multi-level construct spanning (1) documentation infrastructure, (2) workflow-level uptake, and (3) coder-level acceptance. Distinguishing these levels clarifies why real-world deployment success cannot be inferred solely from model accuracy: reliable performance depends on the structural properties of the documentation ecosystem, while sustained operational use depends on implementation conditions and user experience. This framing provides a practical template for reporting and interpreting adoption in real-world clinical AI evaluations, where the term is frequently invoked but inconsistently defined.

Consistent with this multi-level perspective, our real-world evaluation demonstrates that AI-assisted ICD-10-CM coding can be feasibly integrated into existing clinical workflows. In the 13-week RCT, the human-in-the-loop workflow substantially reduced time-to-code while maintaining high coding accuracy under routine workload fluctuations, alleviating part of the cognitive and administrative burden historically borne by CCSs. These gains were enabled not only by model performance but also through coordinated institutional support, including department leadership endorsement, collaboration with the hospital’s information technology team, and targeted user training. Workflow-level adoption increased steadily from 37.26% to 90.59% over the study period, indicating that with structured onboarding, CCSs can rapidly develop operational familiarity and trust. Measuring adoption in billing-driven environments introduces additional complexity beyond typical task-level usage metrics. Utilization signals may be shaped by claim and documentation workflows (e.g., interim monthly claims for long-stay admissions), such that a single patient trajectory may generate multiple coding episodes, inflating or deflating apparent uptake depending on operational cadence. Accordingly, we interpreted the adoption rate reported here as a workflow-level utilization indicator rather than a direct measure of task-level update. These results illustrate how well-chosen models can translate into tangible workflow benefits when paired with thoughtful implementation strategies.

At the individual level, satisfaction analyses revealed substantial heterogeneity in how CCS subgroups experienced the AI-assisted workflow. Contrary to our H2b (Supplementary Note 8), which hypothesized higher satisfaction among less experienced CCSs based on technology readiness and innovation diffusion theory^36–38^, junior CCSs with fewer than 10 years of experience reported lower satisfaction than senior coders, and generational trends followed a U-shaped pattern, with Gen X and Gen Z reporting higher satisfaction than Gen Y, rather than the expected monotonic increase from Gen X to Gen Z owing to “digital native”^39^ (H2b). One possible interpretation is that operational trust reflects the alignment between AI recommendations and established professional workflows, as well as perceived role security and responsibility for final coding decisions, rather than age or technical familiarity per se^40^. These patterns suggest that domain expertise, generational cohort, and career stage jointly act as important moderators of AI acceptance. Experienced CCSs may benefit from using the AI outputs as a verification aid, whereas junior CCSs may experience uncertainty when interpreting or validating model suggestions. Late-career considerations also influenced adoption, as illustrated by the withdrawal of a near-retirement CCS, which is consistent with prior work’s report that noted workers nearing retirement may perceive limited personal benefits from investing in new digital tools and may prioritize stability over experimentation^41^. Together, these findings highlight that successful human–AI integration depends on role-sensitive onboarding, experience-aware support, and designs that reinforce coder autonomy and responsibility.

Furthermore, our findings highlight the importance of broader equity considerations in AI-assisted coding across heterogeneous clinical environments. Although both participating hospitals used HL7-CDAR2 documentation infrastructure, differences in written guidelines, documentation completeness, and section emphasis still produced variability that affected section-wise models more strongly than universal modeling. These observations emphasize that institutional documentation practices, data availability, and workflow conventions can introduce systematic variability in model reliability, potentially disadvantaging settings with less structured or incomplete notes. Ensuring equitable AI performance across hospitals will therefore require attention not only to model robustness but also to documentation standardization, input completeness, and upstream workflow variability. Future work should expand longitudinal post-deployment monitoring, explore adaptive update mechanisms to mitigate model drift, investigate coder-AI interaction strategies tailored to experience levels, and examine the operational effects of AI assistance on billing integrity and concurrent inpatient coding workflows.

Finally, several limitations of this approach should be acknowledged. Although pairwise LLM-based evaluation provides a scalable proxy for assessing definition-level intrinsic semantic competence, the judge model itself is not a source of ground-truth clinical authority and may reflect biases inherited from its training data or prompting context. Our framework, therefore, does not assume perfect judge accuracy; instead, it relies on relative comparisons, aggregation across many judgments, and downstream empirical validation. In particular, the intrinsic definition-generation task is framed as a pragmatic screening heuristic rather than a causal or theoretical guarantee of downstream ICD-10-CM coding ability. Moreover, the observed alignment between intrinsic rankings and downstream coding performance was demonstrated on a limited set of candidate models in this study and should not be interpreted as establishing a causal relationship between definition generation quality and ICD-10-CM coding ability.

While the strong alignment between intrinsic rankings and downstream coding performance supports the practical, resource-aware utility of this approach as a screening heuristic, future work should further examine the behavior of different judge models, incorporate limited expert adjudication where feasible, and explore hybrid evaluation strategies that combine automated and human-in-the-loop assessment. Evaluating multi-judge ensembles or measuring agreement across heterogeneous judges represents a particularly important direction for strengthening confidence in automated model selection. More broadly, assessing this selection strategy on a larger and more diverse pool of foundation models, as well as across additional institutions, languages, and time periods, will be crucial to better characterize its generalizability and failure modes under evolving clinical documentation and coding standards.

Additionally, our error analysis suggests that a meaningful signal of failure modes emerges from real-world factors that are not fully captured by aggregate performance metrics. First, we observed cases where CCSs assigned ICD-10-CM codes that were not present in the training corpus (e.g., Z86.16 for personal history of COVID-19 and other emerging or infrequent codes). In these instances, models failed to generate the correct codes, reflecting limitations in label coverage rather than semantic misunderstanding. Such out-of-vocabulary cases were more evident in cross-institutional evaluation, where regional practice patterns (southern vs. eastern Taiwan) and temporal differences between training (2019-2021) and evaluation periods introduced systematic mismatches, particularly for codes that became salient later (e.g., COVID-related conditions). This mismatch is consistent with the differing ICD-10-CM chapter distributions observed across external datasets (Supplementary Fig. 2). Second, among in-vocabulary errors, even the best-performing model occasionally exhibited fine-grained confusions, such as misassignment of anatomical sites within related code families and occasional over-generation of nonspecific symptom codes when symptom mentions co-occurred with more specific etiologies. Third, cross-institution errors were also shaped by documentation conventions: KMUH clinical documentation more consistently concentrates codable diagnoses in DischgDiag, whereas TMMH documentation places diagnostically relevant cues more diffusely across sections, increasing truncation and retrieval sensitivity under fixed token-length constraints. This is particularly salient for TMMH’s high-prevalence Z-codes (Supplementary Fig. 2), which are often implied by chief complaints, treatment intent, or care plans rather than explicitly listed as discharge diagnoses. This discrepancy reflects an explicit design trade-off informed by KMUH documentation practice, rather than a modeling error, and highlights how section selection strategies interact with institution-specific coding conventions.

In addition, the pragmatic week-by-week rotation design may introduce residual temporal confounding (e.g., seasonal workload variation or staffing changes) that is not fully separable from workflow assignment. Although the coding interface was deployed and iteratively refined well before AI activation, our primary analyses did not explicitly model week index or time-on-study as covariates. Accordingly, time-to-code estimates should be interpreted as operational effects under routine clinical variability rather than as fully de-confounded causal estimates. Future pragmatic deployments could strengthen attribution by incorporating temporal covariates and using stepped-wedge or block-randomized designs to examine time trends under stable operational conditions.

Importantly, the early termination of the RCT due to a nationwide ICD-10-CM version transition highlights a practical constraint of real-world clinical AI deployment: experimental protocols cannot override regulatory or reimbursement-driven system changes. This disruption, while limiting full crossover balance, reflects the operational reality that clinical AI systems must remain robust to evolving coding standards, policy updates, and infrastructure changes. This underscores the importance of developing AI-assisted coding systems that can accommodate evolving coding standards and policy updates, a consideration that is rarely captured in controlled benchmarking studies.

Together, these observations suggest that improving real-world reliability will require not only stronger model architectures but also strategies for continual vocabulary expansion, institution-aware calibration, and post-deployment monitoring to detect performance decay under evolving coding practices.

## Methods

### Dataset

This study was conducted in accordance with the Declaration of Helsinki and approved by the relevant institutional review boards (IRBs) of the participating institutions. Ethical approval was obtained from KMUH under approval numbers KMUHIRB-E(II)-20230214 (“Developing Artificial Intelligence Model to Support ICD-10-CM/PCS Coding and Comparing the Performance between Machine Coding and Manual Coding”), from National Health Research Institutes under approval number EC1140210-E (“Multimodal AI Technologies for Optimized Applications and Comprehensive Deployment in Healthcare”), and from TMMH under approval number 24MMHIS413e (“Implementation Plan for Smart Healthcare ICD-10-CM/PCS Coding Prediction Model”). We assembled three anonymized, ICD-10-annotated clinical datasets from two collaborating hospitals, all formatted according to the HL7-CDAR2 standard. In contrast to public corpora such as MIMIC-III, which primarily contain unstructured free text and are limited to ICD-9 codes, these datasets provide ICD-10-CM/PCS codes and HL7-aligned document structure, enabling section-aware modeling in downstream experiments. All datasets used for model development and performance evaluation were retrospectively collected and fully de-identified prior to analysis. The requirement for informed consent was waived by the approving ethics committees due to the retrospective nature of the study, the use of anonymized data, and the absence of any direct patient contact.

In consultation with CCSs, five key sections most relevant to diagnostic and procedural code assignments were selected for inclusion in the compiled dataset: DischgDiag, MedHist, OpNote, PathRep, and TreatCous. These sections formed the standardized input schema for all modeling experiments. Examples of de-identified sectioned documents are provided in Supplementary Table 4. The final column of the table presents the section-checking priority rankings as recommended by CCSs, reflecting the relative importance of each section during manual review for ICD-10 code assignment.

All datasets underwent a unified data cleaning procedure described in Supplementary Note 1, including duplicate removal, consistency checks, and normalization. We applied multi-label stratified sampling^42^ (8:1:1 split) to preserve ICD-10 label distribution across the training, development, and test sets. Because our LLM experiments were constrained to a 2048-token limitation, priority-based truncation was applied when necessary: lower-priority sections, as ranked by CCSs, were removed first to retrain diagnostically essential content in the input.

### Redundancy-aware sampling strategy

To reduce overfitting and improve training efficiency, we developed a redundancy-aware sampling method using sentence embeddings and approximate nearest neighbor search^43^ to identify and remove semantically repetitive discharge summaries that shared identical ICD-10-CM codes, which offer limited incremental learning value. Specifically, each discharge summary was encoded into a semantic vector using the pretrained all-MiniLM-L6-v2 sentence transformer^44^. A FAISS index^45^ was then constructed to facilitate fast vector search, and L2 distances between embeddings were computed as proxies for semantic similarity. For each summary, its nearest semantic neighbor was identified. If both records shared identical ICD-10 codes and their semantic similarity exceeded a threshold $\tau$ (set to 0.9 to target only highly similar duplicates under identical code sets; robustness was assessed by repeating the pipeline with $\tau \in \{\mathrm{0.85,0.9,0.95}\}$, with resulting removal rates and downstream performance summarized in Supplementary Table 12), they were marked as redundant and subject to removal.

To determine which summary to retain from each redundant pair, we compute the perplexity (PPL) of both candidates using the selected base LLM. If one summary exhibited a PPL at least 5% higher than its counterpart—indicating greater linguistic uncertainty and potentially more diverse language—it was retained. If PPLs were within the threshold, the longer summary was preserved to retain more contextual information.

### Pretrained base model selection

Selecting an appropriate pre-trained base model is a critical step in developing a robust and reliable medical coding system, as general-purpose LLMs often demonstrate limited grounding in ICD taxonomies^11–13^. Rather than assuming that downstream fine-tuning alone can compensate for such gaps, we proposed a model-agnostic intrinsic evaluation framework to screen candidate models based on their definition-level handling of ICD-10-CM concepts prior to task-specific adaptation. Five decoder-only LLMs with fewer than 7 billion (B) parameters were selected based on deployment feasibility within hospital IT environments and prior benchmarking evidence^12^. The candidate models included PubMedGPT-2, which was utilized in our previous work^8^, Llama2-7B, Mistral-7B instruct, along with their domain-adapted variants MedLlama2 and BioMistral-7B.

Rather than manually comparing textual outputs of candidate LLMs to reference answers^30^, or relying on surface-/semantic-level metrics such as lexical overlap^12^ or cosine similarity^11^, we formulated base model selection as a pairwise preference task based on LLM-as-judge^24^. For each of the top 50 most frequent ICD-10-CM codes in our training corpus, two candidate models were prompted to generate clinical descriptions of the target code. A lightweight LLM (Atla Selene Mini^46^) served as an automated judge, independently determining which output demonstrated higher semantic fidelity and clinical appropriateness. Irregular judge outputs were normalized using pattern-based rules, and ambiguous cases were recorded as ties. The prompt template used by the judge model is shown in Supplementary Table 13.

Based on the resulting pairwise comparisons, we construct a directed comparison graph to represent the matchup results among them. Following Luce^25^’s choice axiom, the pairwise comparison process yields a Plackett-Luce model, which can infer a global preference ordering from pairwise data based on its stationary distribution^47^. The distribution can be estimated by using the iterative Luce spectral ranking algorithm^48^, and the resulting selection probabilities were used as a heuristic criterion for identifying a high-potential base model, rather than as an absolute measure of model capability. The model with the highest selection probability was designated as the base model for downstream ICD-10-CM coding task fine-tuning.

This approach enables pragmatic, resource-efficient model screening without exhaustive fine-tuning of all candidates, and remains robust even when only partial or incomplete comparison matrices are available. Importantly, this intrinsic evaluation is intended as a deployment-aware selection heuristic rather than a claim of causal linkage between definition-generation ability and downstream ICD-10-CM coding performance. Full prompt templates, judge variants, mathematical formulation, and implementation details are provided in Supplementary Note 9.

### Section-wise instruction turning

**Fig. 9:**
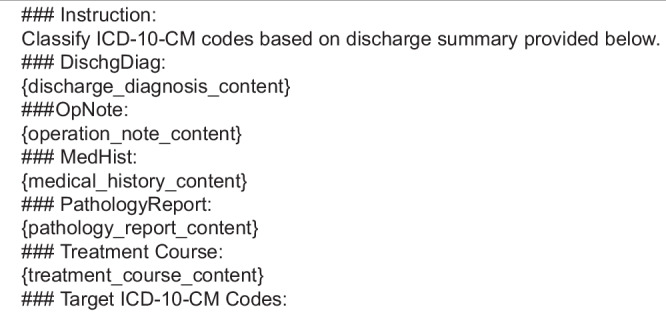
Instruction tuning template with section-wise Health Level Seven Clinical Document Architecture Release 2 (HL7-CDAR2) information for the International Classification of Diseases, Tenth Revision, Clinical Modification coding task. Braces represent placeholders for the exact content for each HL7-CDAR2-compliant section.

After selecting the optimal base model, we applied a section-wise instruction tuning approach as part of supervised parameter-level fine-tuning, rather than prompt-only inference, to incorporate structured HL7-CDAR2 content into the training process. Specifically, the instruction-style templates were used to define the input-output structure during training, while model parameters were updated via gradient-based optimization to learn the ICD-10-CM coding task. Each discharge summary was reformatted using an instruction-style section-wise prompt template (Fig. 9) in which clinical sections (*e.g*., DischgDiag) were explicitly marked with the prefix “###”, followed by the section name and its content. This template, adapted from the design principles of Bsharat, et al.^49^ reinforces semantic segmentation within the clinical text and enables the model to better distinguish diagnostically relevant information from procedural or historical context, which serves to align parameter learning with clinically meaningful document structure, rather than to study prompt sensitivity.

To examine the influence of section-wise content inclusion, we fine-tuned two model families: (1) The universal section-aware model was trained using the full template in Fig. 9, with missing sections explicitly filled with the “Nil” token during both training and inference, enabling the model to operate robustly across heterogeneous documentation patterns commonly observed in real-world deployments. (2) Section-combination-specific models. Each model in this family was tine-tuned on a restricted subset of sections (e.g., DischgDiag+MedHist) using similar prompt template but includes only the specific sections relevant to that section subset. All other sections—including their headers—were entirely omitted to reflect the intended structural scope. Required sections that were absent in the raw summary were also omitted to maintain strict structural alignment during training and inference.

All models were trained to generate ICD-10-CM predictions using a structured output format that distinguishes the primary diagnosis code from other codes, reflecting standard clinical coding practice and supporting downstream reimbursement workflow. The complete supervised fine-tuning objective, including the structured output format and the mathematical formulation of the autoregressive training loss, is provided in Supplementary Note 10. Importantly, all other baseline models—including BiGRU, HAN, BERT, and PLM-ICD—shown in Table 1 were trained and evaluated using the same training, validation, and test splits as the decoder-based models, rather than being directly adopted from prior literature.

### Experimental design: pilot RCT

To evaluate the real-world utility of AI-assisted ICD-10-CM coding within routine hospital workflows, we conducted a 13-week pilot RCT at KMUH. This pragmatic, human-in-the-loop study was designed to assess both workflow efficiency and user experience under naturalistic clinical conditions. The trial addressed two RQs outlined in the Introduction section, focusing on whether AI assistance improved coding efficiency (RQ1) and how model type and coder characteristics shape user satisfaction (RQ2). Based on these objectives, we defined three hypotheses prior to study initiation:

CCSs assigned to AI-assisted workflows, as shown in Supplementary Fig. 3, will complete coding tasks more efficiently than those in the control (manually coding) workflow^50^.

- Model type significantly affects satisfaction, with the domain-specialized BioMistral model expected to yield higher perceived usability than HAN and PubMedGPT-2.
- CCS’s background characteristics influence satisfaction with AI-assisted workflows.

Rather than subdividing H2b into multiple narrowly defined hypotheses, CCS background characteristics—including years of experience^36–38^, certification level^51^, education background^52^, and generation cohort^39^—were treated as exploratory moderators. This design choice reflects both the pilot nature of the study and the expected interdependence among user characters. The conceptual rationale for this approach is detailed in Supplementary Note 8.

Eleven CCSs participated in the trial between October and December 2024. A week-by-week rotation-based randomization schedule (see Supplementary Table 14) was used to assign each CCS to one of four workflows: non-AI-assisted (control group) and three AI-assisted coding workflows (experimental groups), including HAN, PubMedGPT-2, and BioMistral across study weeks. All participants rotated through every workflow during the study period, but weekly assignments varied across CCSs and model configurations. As a result, individual coding sessions were analyzed as independent observations rather than paired measurements. For each coding task, coding time was automatically recorded from the activation of the AI-assisted coding interface to task completion (defined as clicking the save button). Workflow-level uptake of the AI-assisted system was tracked throughout the study period; the operational definition and computation of adoption metrics are detailed in Supplementary Note 11.

To capture real-time user experience, a 5-point Likert satisfaction survey (Supplementary Fig. 4) was randomly triggered during coding curation. Coding time was automatically recorded, and satisfaction surveys were triggered randomly, linked to the corresponding workflow condition, and collected anonymously to mitigate response bias. Note that due to the workflow-embedded nature of AI assistance, blinding to the presence of AI was not feasible. However, CCSs were blinded to the identity of the underlying AI model (HAN, PubMedGPT-2, or BioMistral) used in each AI-assisted workflow. Note that the planned rotation schedule was partially truncated due to an institution-wide ICD-10-CM version transition, resulting in approximately—but not perfectly—balanced model exposure across CCSs; details are provided in Supplementary Table 14.

To evaluate RQ1, coding time outliers were excluded by trimming the top and bottom 2.5% of observations. Welch’s ANOVA with Games–Howell post hoc tests was used to compare mean coding time across workflows. For RQ2, we applied multinomial logistic regression to examine the association between satisfaction levels and both model type and CCS background characteristics. All statistical analyses were conducted in SPSS version 21 using a significance threshold of *p* < 0.05.

## Supplementary information

Supplementary information

## Data Availability

The datasets generated and/or analyzed during the current study are not publicly available due to ethical and legal restrictions related to patient privacy and IRB approval. The data contain potentially identifiable clinical information derived from electronic health records and clinical coding workflows, and therefore cannot be shared openly. No public or third-party access to the data is permitted under the approved ethical protocol.

## Code availability

The code used for model training, evaluation, and analysis is available from the corresponding author upon reasonable request.
