# Supplementary material for: Evaluating real-world deployment of an HL7-CDA-aligned LLM for ICD-10-CM coding
Source: NPJ Digit Med. 2026 Apr 14;9:561. doi: 10.1038/s41746-026-02541-5 (PMC13385930; doi:10.1038/s41746-026-02541-5)
Supplement: Supplementary file 1 — Supplementary information [file 41746_2026_2541_MOESM1_ESM.pdf]

Supplementary Information

Supplementary Tables

Supplementary Table 1. Full list of HL7-CDA-release 2 sections defined in the collected discharge summary dataset.

Section	Used in this study
Subjective	
Admission Diagnosis	
Discharge Diagnosis	✓
Medical History	✓
Physical Exam	
Treatment Course	✓
Pathology Report	✓
Exam Report	
Lab Test Results	
Radiology Report	
Operation Note	✓
Discharge Status	
Combined Complications	
ICD-10-CM Code	✓

The discharge summary used in this study follows the Health Level Seven (HL7) Clinical Document Architecture (CDA) release 2 format, segmented into fourteen predefined sections. These sections capture various aspects of the patient encounter, ranging from concise entries such as "Subjective" to more comprehensive ones like "Admission Diagnosis", "Medical History", and "Discharge Diagnosis".

Supplementary Table 2. Distribution of section combinations in the KMUH dataset.

Section Combination	Reports Containing Combination (Test) / Report Exclusively Containing Combination (Test)	Reports Containing Combination Exceeding 2,048 tokens (%)
(1) Discharge Diagnosis	125,820 (12,582) / 0 (0)	0
(2) 1 + Medical History	124,624 (12,463) / 35 (4)	12.89
(3) 1 + Operation Note	86,376 (8,638) / 0 (0)	12.60
(4) 3 + Medical History	86,341 (8,635) / 6 (1)	12.60
(5) 4 + Pathology Report	65,514 (6,552) / 55 (6)	14.04
(6) 5 + Treatment Course	65,459 (6,546) / 65,459 (6,546)	14.03

Each row represents a unique combination of discharge summary sections found in the training and test datasets. The second column indicates both the number of summaries containing the combination and the number of summaries that exclusively contain that specific combination. The third column reports the

percentage of summaries within each combination that exceed 2,048 tokens in length, based on tokenized input.

Supplementary Table 3. Distribution of section combinations in the Taitung MacKay Memorial Hospital (TMMH) test dataset.

Section Combination	Reports Containing Combination	/	Report Exclusively Containing Combination
(1) Discharge Diagnosis	33,122	/	0
(2) 1 + Medical History	33,104	/	0
(3) 1 + Operation Note	9,287	/	0
(4) 3 + Medical History	9,287	/	0
(5) 4 + Pathology Report	3,819	/	0
(6) 5 + Treatment Course	3,819	/	3,819

Each row represents a unique combination of discharge summary sections found in the test dataset. The second column indicates both the number of summaries containing the combination and the number of summaries that exclusively contain that specific combination. Note that the lengths of all summaries are less than 2,048 tokens.

Supplementary Table 4. A deidentified example of a discharge summary conforming to HL7-CDA Release 2 standards used in this work.

Section Name	Section Text	Suggested Priority
Discharge Diagnosis	1. Right distal radius fracture<br>- post open reduction and internal fixation of right distal radius fracture on 2043/6/2<br>--- Underlying diseases ---<br>. Left hydronephrosis, status post double-J insertion on 2041/09/25, 2042/7/14<br>. Recurrent cervical cancer with metastatic left iliopoas muscle adenocarcinoma …	1

Medical History	■HPI (History of Present Illness):<br>Patient was a 67-year-old woman with underlying disease of:<br>. Left hydronephrosis status post double-J insertion on 2031/09/25, 2042/7/14<br>. Recurrent cervical cancer with metastatic left iliopoas muscle adenocarcinoma<br>…<br>She fell down on 5/30 in morning resulting in right wrist painful disability. She was brought to our emergent room and then …<br>■PH (Past History): + Past History<br>DM (diabetes mellitus): denied<br>Hypertension: +<br>CKD (chronic kidney disease): denied	
Pathology Report	no exam during admission.	4
Operation Note	Open reduction and internal fixation of right distal radius fracture on 2043/6/2	2
Treatment Course	After admission, this 73-year-old female received our general survey. She underwent Open reduction and internal fixation of right forearm smoothly on 2043/6/2, and no peri-operative complications were noted. She had right forearm operation pain (Visual Analogue Scale:3/10). We gave her Cefazolin and …	5

Only the recommended sections shown in Supplementary Table 1 are included in the example.

Supplementary Table 5. ICD-10-CM coding performance comparison across HL7-CDAR2 section-wise content combinations for both section combination-specific and universal BioMistral models.

Model Type	Included Sections	Full Test Set			Section Combined Test Subset		
		P	R	F	P	R	F
Section-combination-specific Model	1) DischgDiag	**0.798**	0.776	0.786	0.798	0.776	0.787
	2) 1) +OpNote	0.773↓	0.754↓	0.764↓	0.773↓	0.754↓	0.764↓
	3) 1) +MedHist	0.807↑	0.782↑	0.794↑	0.807↑	0.782↑	0.794↑
	4) 2) +MedHist	0.793↓	0.773↓	0.783↓	**0.807**↑	0.786↑	0.796↑
	5) 4) +PathRep	0.779↓	0.792↑	0.785↓	0.801↑	0.795↑	0.798↑
	6) 5) +TreatCous	0.796↓	**0.800**↑	**0.798**↑	0.800↑	**0.801**↑	**0.800**↑
Universal Model	1) DischgDiag	0.764	0.766	0.765	0.763	0.766	0.764
	2) 1) +OpNote	0.773↑	0.733↓	0.752↓	0.769↑	0.758↓	0.763↓
	3) 1) +MedHist	0.797↑	0.772↑	0.784↑	**0.802**↑	0.776↑	0.789↑
	4) 2) +MedHist	0.802↑	0.775↑	0.788↑	**0.801**↑	0.791↑	0.796↑
	5) 4) +PathRep	0.801↑	0.778↑	0.789↑	0.800↑	0.795↑	0.797↑
	6) 5) +TreatCous	**0.808**↑	**0.796**↑	**0.802**↑	0.800↑	**0.801**↑	**0.800**↑

Each row corresponds to a unique training section combination. For the universal model, the same model is

reused across all evaluations with only matched section content retained per test case. The direction of the arrow in each cell indicates the relative performance change compared to the baseline model trained solely on the DischgDiag section: ↑ indicates improvement, ↓ indicates decline, and → indicates no change.

Supplementary Table 6. Demographic characteristics of the CCSs who participated in the KMUH RCT.

**Variable**	**Number, n/N(%)**
Coding experience	
1-10 years	7, 0.7(70)
11-24 years	2, 0.2(20)
25+ years	1, 0.1(10)
Generation	
Generation X (1965-1980)	4, 0.4(40)
Generation Y (1981-1996)	4, 0.4(40)
Generation Z (1997-2012)	2, 0.2(20)
Education	
Medical-related Programs	3, 0.3(30)
Health/Medical Administration Programs	7, 0.7(70)
Certificates	
Advanced CCS	1, 0.1(10)
CCS	9, 0.9(90)

The randomized controlled trial (RCT) was conducted over a 13-week period in Kaohsiung Medical University Chung-Ho Memorial Hospital (KMUH), from October to December 2024, covering a total of 91 days with a randomization schedule, as shown in Supplementary Table 6.

Supplementary Table 7. Multinomial logistic regression of satisfaction levels by model type among CCSs. (reference: BioMistral & Very Dissatisfied).

**Satisfaction**	**Variable**	**OR (95% CI)**	***P* value**
Dissatisfied	HAN	0.383 (0.236-0.621)	$< 0.01^{*}$
	PubMedGPT2	0.651 (0.366-1.155)	0.142
Neutral	HAN	0.166 (0.103-0.266)	$< 0.001^{*}$
	PubMedGPT2	0.485 (0.280-0.840)	0.010^{*}
Satisfied	HAN	0.135 (0.085-0.214)	$< 0.001^{*}$
	PubMedGPT2	0.496 (0.290-0.848)	0.010^{*}
Very Satisfied	HAN	0.203 (0.109-0.376)	$< 0.001^{*}$
	PubMedGPT2	0.506 (0.800-1.547)	0.506

Odds ratios (OR) < 1 indicates reduced likelihood of belonging to the specified satisfaction category compared with the reference combination. $P < 0.05$ is considered statistically significant. Regression reference:

BioMistral model and “Very Dissatisfied” category.

Supplementary Table 8. Multinomial logistic regression of satisfaction levels by seniority among CCSs. (reference: Seniority >25 years & Very Dissatisfied).

Satisfaction	Variable	OR (95% CI)	*P* value
Dissatisfied	<10 years	0.020 (0.003-0.143)	< 0.001*
	10-25 years	0.032 (0.003-0.404)	0.008*
Neutral	<10 years	0.048 (0.007-0.349)	0.003*
	10-25 years	0.788 (0.070-8.916)	0.848
Satisfied	<10 years	0.019 (0.003-0.134)	< 0.001*
	10-25 years	1.514 (0.136-16.860)	0.736
Very Satisfied	<10 years	0.008 (0.001-0.056)	< 0.001*
	10-25 years	0.019 (0.001-0.300)	0.005*

The model includes seniority only; no additional covariates were adjusted. Reference groups include “Seniority >25 years” and “Very Dissatisfied”. OR < 1 indicates reduced odds of belonging to a higher satisfaction category. *P* < 0.05 indicates statistical significance. Given the extremely small size of the reference group (*n* = 1 for >25 years), the estimates may be unstable and should be interpreted with particular caution. In addition, subgroup distributions are highly unbalanced across seniority levels (<10 years: *n* = 7; 10-25 years: n = 2; >25 years: *n* = 1), which may inflate standard errors and limit the generalizability of the results.

Supplementary Table 9. Multinomial logistic regression of satisfaction levels by CCS certification level among CCSs. (reference: Advanced CCS & Very Dissatisfied).

Satisfaction	Variable	OR (95% CI)	P value
Dissatisfied	Standard CCS	0.020 (0.003-0.144)	< 0.001*
Neutral	Standard CCS	0.057 (0.008-0.417)	0.005*
Satisfied	Standard CCS	0.037 (0.005-0.270)	0.001*
Very Satisfied	Standard CCS	0.008 (0.001-0.057)	< 0.001*

The model includes certification level only; no covariates were adjusted. Reference groups include “Advanced CCS” and “Very Dissatisfied”. OR < 1 indicates reduced odds of belonging to a higher satisfaction category. The estimates should be interpreted cautiously due to unbalanced group sizes. *P* < 0.05 indicates statistical significance. Given the unbalanced distribution of participants, with a disproportionate number of CCSs holding “Standard CCS” certification (*n* = 9) compared to “Advanced CCS” (n = 1), the estimates should be interpreted with caution. Small group sizes, particularly the reference category, may inflate standard errors and reduce the stability of the estimated odds ratios.

Supplementary Table 10. Multinomial logistic regression of satisfaction levels by medical background among CCSs. (reference: Medical Management Background & Very Dissatisfied).

**Satisfaction**	**Variable**	**OR (95% CI)**	***P* value**
Dissatisfied	Medical-major CCSs	0.025 (0.010-0.062)	< 0.001*
Neutral	Medical-major CCSs	0.013 (0.005-0.032)	< 0.001*
Satisfied	Medical-major CCSs	0.009 (0.004-0.023)	< 0.001*
Very Satisfied	Medical-major CCSs	0.030 (0.012-0.079)	< 0.001*

The model includes CCS education background only; no covariates were adjusted. Reference groups include “a major in medical management” and “Very Dissatisfied”. OR < 1 indicates reduced odds of belonging to a higher satisfaction category. The estimates should be interpreted cautiously due to unbalanced group sizes. *P* < 0.05 indicates statistical significance.

Supplementary Table 11. Multinomial logistic regression of satisfaction levels by generational cohorts among CCSs. (reference: Gen Y & Dissatisfied).

**Satisfaction**	**Variable**	**OR (95% CI)**	***P* value**
Neutral	Gen X	1.961 (1.484 to 2.592)	< 0.001*
	Gen Z	2.137 (1.535 to 2.975)	< 0.001*
Satisfied	Gen X	12.523 (9.449 to 16.597)	< 0.001*
	Gen Z	6.010 (4.277 to 8.446)	< 0.001*

The model includes generational cohort only; no covariates were adjusted. Reference groups include “Gen Y” and “Dissatisfied” (combined with “Very Dissatisfied”). OR < 1 indicates reduced odds of belonging to a higher satisfaction category. The estimates should be interpreted with caution due to potential residual confounding and unbalanced group sizes. *P* < 0.05 indicates statistical significance.

Supplementary Table 12. Sensitivity analysis of redundancy-aware sampling with respect to the semantic similarity threshold τ.

Redundant-aware Sampling Threshold (τ)	F-Measure	Removal Rate
Original	0.7797	-
$\tau = 0.95$	0.7848	0.037
$\tau = 0.90$	0.7868	0.161
$\tau = 0.85$	0.7808	0.192

Supplementary Table 13. Prompts used for two candidate LLMs and the judge LLM.

Role	Prompt
Candidate LLMs	As an ICD-10-CM coder, I need to know the definition of the provided ICD-10 code. Here is the provided code: [ICD-10-CM CODE].
Judge LLM	You are a helpful assistant in evaluating the quality of the responses for a given instruction. Your goal is to select the best response for the given instruction. Select Response A or Response B, that is better for the given instruction. The two responses are generated by two different AI ICD-10 coding agents respectively. Do NOT say both / neither are good.<br>Here are some rules of the evaluation:<br>(1) You should prioritize evaluating whether the response satisfies the provided rubric. Then consider its helpfulness, accuracy, level of detail, harmlessness, etc.<br>(2) Responses should NOT contain more/less than what the instruction asks for, as such responses do NOT precisely execute the instruction.<br>(3) You should avoid any potential bias and your judgment should be as objective as possible. Here are some potential sources of bias:<br>- The order in which the responses were presented should NOT affect your judgment, as Response A and Response B are **equally likely** to be the better.<br>- The length of the responses should NOT affect your judgement, as a longer response does not necessarily correspond to a better response. When making your decision, evaluate if the response length is appropriate for the given instruction.<br>Your reply should strictly follow this format:<br>**Reasoning:** <feedback evaluating the responses><br>**Result:** <A or B><br>Here is the data.<br>Instruction:<br>```<br>{user_input}<br>```<br>Response A:<br>```<br>{assistant_response_a}<br>```<br>Response B:<br>```<br>{assistant_response_b}<br>```<br>Score Rubrics:<br>Score 1: Incorrect or unrelated response<br>- Response is empty, irrelevant, hallucinated, or completely wrong.<br>- No meaningful connection to the correct ICD definition.<br>Example: {example_1}<br>Score 2: Mostly incorrect, with minimal relevance

- Describes an unrelated or incorrect condition.

- May use similar medical terms but is fundamentally wrong.

Example: {example_2}

Score 3: Partially correct, but contains vague or mixed concepts

- Contains some relevant concepts (e.g., mentions related symptoms or illness).

- But lacks clarity or introduces ambiguous/incorrect terminology.

- User may need to double-check or could be misled.

Example: {example_3}

Score 4: Mostly correct with minor omissions or simplification

- Definition is accurate but lacks specificity or formal wording.

- Still clearly reflects the meaning of the ICD code.

- May omit terms like "essential" or "primary".

Example: {example_4}

Score 5: The response is completely accurate and contextually correct.

- Clearly and precisely defines the ICD-10 code.

- Matches the official definition (e.g., from CDC or WHO).

- Includes clinical qualifiers like "essential", "primary", etc. if required

- No factual errors or ambiguity.

Example: {example_5}

Reference answer:

{reference_response}

Supplementary Table 14. The blind rotation-based randomization schedule used in the pilot RCT to assign AI-assisted coding models across CCSs. C0 denotes the controlled workflow without AI assistance; AI1, AI2, and AI3 correspond to the HAN, PubMedGPT-2, and BioMistral models, respectively. Gray-shaded cells indicate the planned schedule weeks that were not executed because the trial was terminated early following an ICD-10-CM version transition, resulting in approximately balanced but not perfectly uniform exposure across CCSs and model conditions.

CCS	Oct. 1	Oct. 8	Oct. 15	Oct. 22	Nov. 5	Nov.12	Nov.19	Nov.26	Dec. 3	Dec. 10	Dec. 17	Dec. 24	Dec.31	Jan. 7	Jan. 14	Jan. 21
A	C0	AI2	AI3	AI1	AI3	AI1	AI2	AI3	AI2	AI1	AI3	AI2	C0	C0	C0	AI1
B	AI1	AI2	AI3	C0	C0	AI1	C0	AI3	AI2	AI1	AI3	AI2	AI3	AI1	AI2	C0
C	AI1	C0	C0	AI1	AI3	C0	AI2	AI3	AI2	AI1	AI3	AI2	AI3	C0	AI2	AI1
D	AI1	AI2	AI3	C0	C0	AI1	AI2	AI3	AI2	AI1	AI3	C0	AI3	AI1	AI2	C0
E	AI1	C0	AI3	AI1	AI3	C0	AI2	AI3	AI2	AI1	AI3	AI2	C0	C0	AI2	AI1
F	AI1	AI2	AI3	C0	AI3	AI1	C0	C0	AI2	AI1	AI3	AI2	AI3	AI1	AI2	C0
G	AI1	AI2	C0	AI1	AI3	AI1	AI2	AI3	AI2	C0	AI3	AI2	AI3	C0	C0	AI1
H	AI1	AI2	AI3	AI1	C0	C0	AI2	AI3	C0	AI1	AI3	AI2	AI3	AI1	AI2	C0
I	C0	C0	AI3	AI1	AI3	AI1	AI2	AI3	AI2	C0	C0	AI2	AI3	AI1	AI2	AI1
J	AI1	AI2	AI3	AI1	AI3	C0	AI2	AI3	AI2	C0	C0	C0	AI3	AI1	AI2	AI1

K	C0	AI2	C0	AI1	AI3	AI1	AI2	AI3	C0	AI1	AI3	AI2	AI3	C0	AI2	AI1

To promote balanced exposure, the three AI-assisted models were assigned using a week-by-week, rotation-based randomization schedule across CCSs during the 13-week pilot period. Because the trial was stopped early due to an ICD-10-CM version transition, model exposure was approximately balanced but not perfectly uniform. Blinding was applied at the model-identity level: CCSs were aware when AI assistance was available, but were not informed which underlying model was activate in the AI-assisted interface shown in Supplementary Figure 3.

Supplementary Figures

Supplementary Figure 1. Distribution of ICD-10-CM (International Classification of Diseases, Tenth Revision, Clinical Modification) chapters in the training, validation, and test sets. The figure presents the statistical information from the primary dataset used for model development and testing. Certain ICD-10-CM chapters appear to have a disproportionately high frequency, particularly chapters I, C, and E, which correspond to diseases of the circulatory system, neoplasms, and endocrine, nutritional, and metabolic diseases. In contrast, chapters such as X (diseases of the respiratory system), Q (congenital malformations, deformations, and chromosomal abnormalities), and Y (external causes of morbidity and mortality) are underrepresented, while chapter U (codes for special purposes such as emergency code additions by the World Health Organization) is absent.

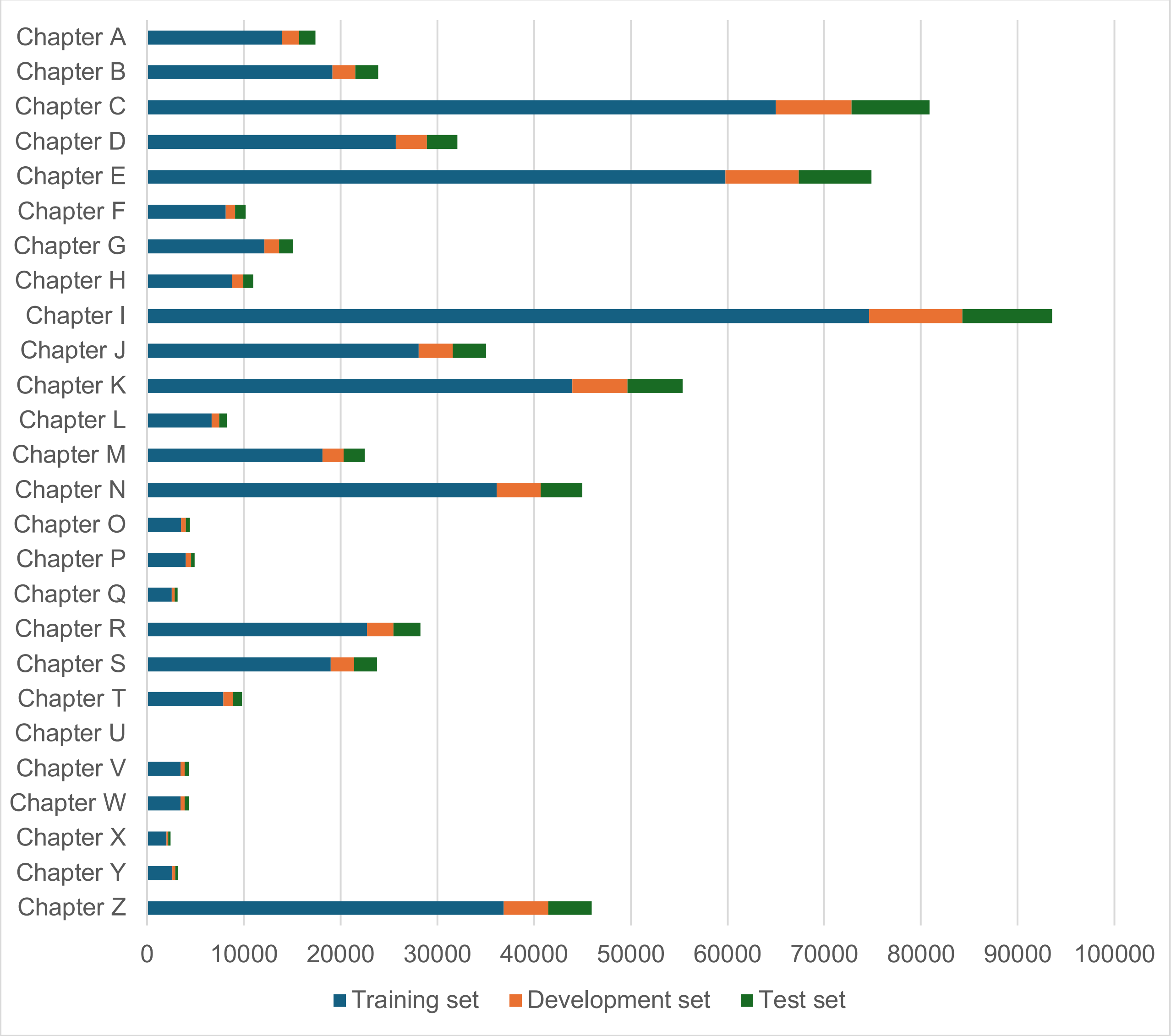

Supplementary Figure 2. ICD-10-CM (International Classification of Diseases, Tenth Revision, Clinical Modification) chapter distribution in the Taitung MacKay Memorial Hospital (TMMH) test set and the Kaohsiung Medical University Chung-Ho Memorial Hospital (KMUH) randomized controlled trial (RCT) test set. The figure compares the chapter-level distribution of ICD-10-CM codes between the TMMU test set and the KMUH RCT test sets. Due to the difference in data collection periods, the two datasets differ significantly in sample size.

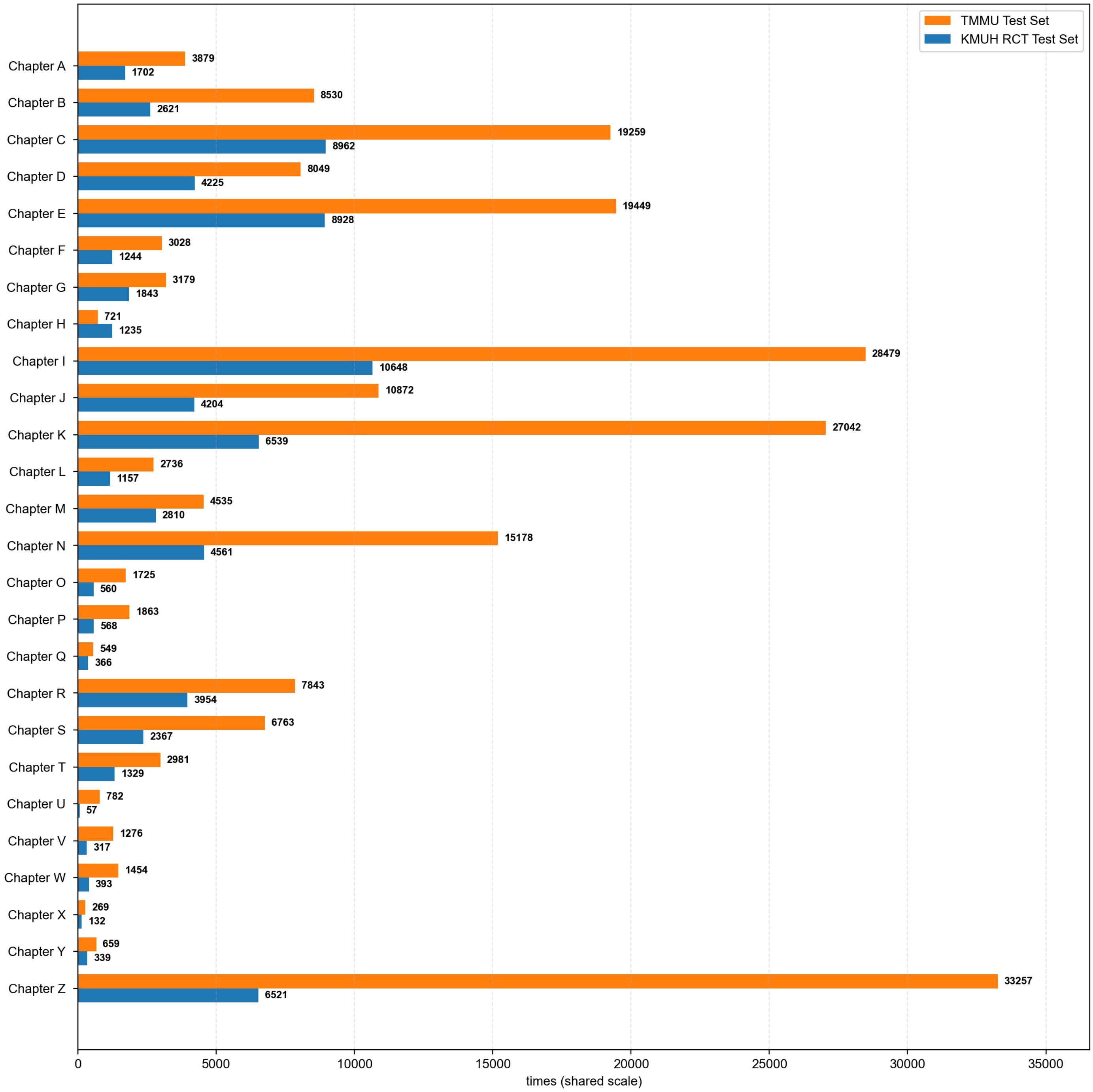

Supplementary Figure 3. User interface of the developed ICD-10-CM (International Classification of Diseases, Tenth Revision, Clinical Modification) coding assistant interface used by Kaohsiung Medical University Chung-Ho Memorial Hospital (KMUH) CCSs. The "Green Box" displays the patient's discharge summary, incorporating all structured sections defined by HL7-CDA Release 2 as outlined in Supplementary Table 1. This narrative content serves as the primary reference for both certified coding specialists (CCSs) and the developed coding model. The "Orange Box" shows the candidate ICD-10-CM codes generated by the underlying coding model. The "Purple Box" is designated for the final codes that have been confirmed and selected (or added) by CCSs following manual review and validation.

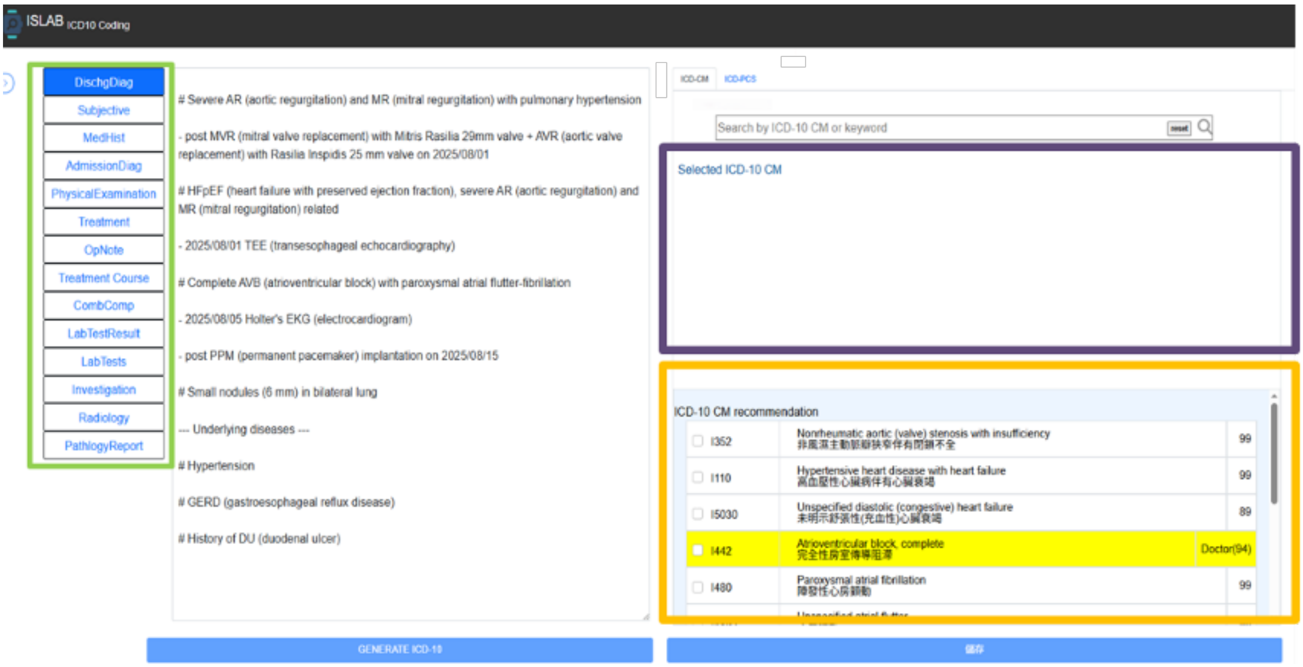

Supplementary Figure 4. The satisfaction survey interface used to collect feedback from certified coding specialists (CCSs) during randomized controlled trial. The survey allows CCSs to rate their overall satisfaction on a 5-point Likert scale (1-5). For cases of dissatisfaction (ratings ≤ 3), users are prompted to specify the reason(s), with predefined options, including the model error rate > 50%, unfriendly user interface, and long response/waiting time. An open text field is also provided to capture additional free-text comments regarding specific issues or suggestions for improvement.

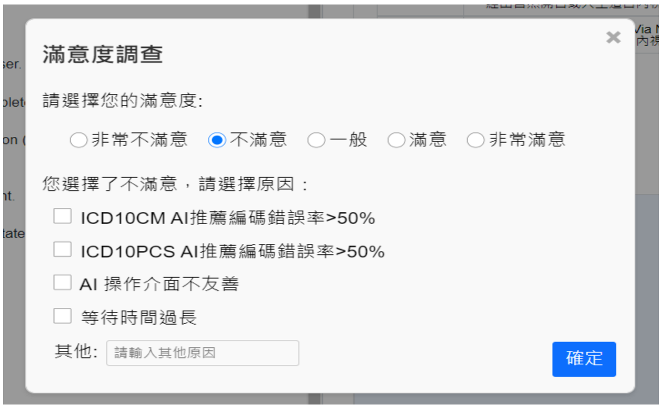

Supplementary Note 1. Data cleaning procedure

Although the internal KMUH and the TMMH datasets were originally curated by CCSs, we observed that some reports either lacked corresponding codes or included incomplete or erroneous entries (e.g., invalid or non-standard ICD codes). To ensure data quality, the following data cleaning procedure was applied. Firstly, discharge summaries missing ICD-10 code assignments or containing invalid code formats were excluded from the dataset. We then applied character-level normalization, standardizing punctuation marks across both Chinese and English texts and removing extraneous whitespaces and line breaks. To retain only clinically relevant content, we applied rule-based pattern matching to strip non-clinical segments, such as administrative notes and timestamped metadata.

For the KMUH RCT test set, due to incomplete procedural data, 1,204 records that lacked timing information were excluded from the analysis. After excluding the top and bottom 2.5% of records with extreme curation times, a total of 10,688 discharge records were retained for analysis.

Supplementary Note 2. Section combination analysis and token length limitation estimation

As shown in Supplementary Table 2, the discharge diagnosis (DischgDiag) section is present in all 125,820 reports, underscoring its central role in discharge summary documentation. The medical history (MedHist) section is the most frequently co-occurring section, with 124,624 reports containing both DischgDiag and MedHist. Of these, 35 contain only these two sections. Other common combinations involve the operation note (OpNote) followed by pathology report (PathRep) and treatment course (TreatCous). Notably, the combination comprising all five sections appears in more than half of reports (65,459), suggesting a standardized documentation structure adopted in a substantial proportion of the dataset.

To assess the feasibility of processing the full contents for model training, we analyzed the token length distribution associated with each section combination. The rightmost column in Supplementary Table 2 presents the percentage of samples in each combination that exceeds the 2,048-token threshold. For instance, 12.89% of summaries in the DischgDiag+MedHist group exceed the limit, compared to 14.03% in the full five-section group. These statistics suggest that setting the maximum input length at 2,048 tokens allows full coverage for approximately 85% of training samples.

Supplementary Note 3. Representative model outputs and LLM-as-judge evaluation results

User Input/Gold definition

I50.9: The definition is Heart failure, unspecified/心臟衰竭

Llama 2 (Response A)

I50 is the code for "Other specified diseases of blood and lymphoid tissue". ICD-CM has a numbering system that allows you to find codes by category, subcategory or even further down into more specificity if needed (either in terms on anatomical location/systems).<br>I50.91A is the code for "Other specified diseases of blood and lymphoid tissue".

Mistral 7B-Instruct (Response B)

I50 is the chapter for circulatory system and ICD-10 code 9 refers to other diseases of heart, which includes: myocardial infarction (MI), angina pectoris without MI or unstable Anginal Equivalents(UAEQ) , cardiac dysrhythmias not elsewhere classified.<br>I50 is further divided into subcategories based on the specific diagnosis within this chapter and ICD-10 code 9 falls under "Other diseases of heart" which includes: other myocardial infarctions, angina pectoris without acute MI or UAEQ (unstable Anginal Equivalents), cardiac dysrhythmias not elsewhere classified.<br>The term 'other' in ICD-10 code 9 refers to any condition that does NOT fall under the more specific codes within this category and is used as a catch all for conditions which do exist but are rare or have insufficient data available at present, such as: other myocardial infarctions (not specified further), angina pectoris without acute MI/UAEQ when no additional information about type of Anginal Equivalents can be determined from the documentation.<br>Therefore ICD-10 code 9 refers to any condition within this category that does not fit into a more specific

subcategory and is used as an alternative for conditions with insufficient data or rare occurrences, such as other myocardial infarctions (not specified further), angina pectoris without acute MI/UAEQ when no additional information about type of Anginal Equivalents can be determined from the documentation.
In summary: ICD-10 code 9 falls under "Other diseases of heart" within chapter I50 and refers to any condition that does not fit into a more specific subcategory, such as other myocardial inf

Judge LLM (Winner: B)

Response A is incorrect as it does not provide the definition of I50.9, which refers to Heart failure unspecified or 心臟衰竭 and also contains irrelevant information about other codes like 91A etc., while response B provides an accurate description but lacks specificity in defining what is meant by 'other' conditions within the category. However, Response A does not provide any relevant definition of I50 which makes it completely incorrect.

BioMistral (Response A)

The ICD-10 code is defined as “Other and unspecified forms of heart failure.” This definition does not match the case scenario, which describes a patient with acute myocardial infarction (AMI).",

Mistral-Instruct (Response B)

I50 is the chapter for circulatory system and ICD-10 code 9 refers to other diseases of heart, which includes: myocardial infarction (MI), angina pectoris without MI or unstable Anginal Equivalents(UAEQ) , cardiac dysrhythmias not elsewhere classified.
I50 is further divided into subcategories based on the specific diagnosis within this chapter and ICD-10 code 9 falls under "Other diseases of heart" which includes: other myocardial infarctions, angina pectoris without acute MI or UAEQ (unstable Anginal Equivalents), cardiac dysrhythmias not elsewhere classified.
The term 'other' in ICD-10 code 9 refers to any condition that does NOT fall under the more specific codes within this category and is used as a catch all for conditions which do exist but are rare or have insufficient data available at present, such as: other myocardial infarctions (not specified further), angina pectoris without acute MI/UAEQ when no additional information about type of Anginal Equivalents can be determined from the documentation.
Therefore ICD-10 code 9 refers to any condition within this category that does not fit into a more specific subcategory and is used as an alternative for conditions with insufficient data or rare occurrences, such as other myocardial infarctions (not specified further), angina pectoris without acute MI/UAEQ when no additional information about type of Anginal Equivalents can be determined from the documentation.
In summary: ICD-10 code 9 falls under "Other diseases of heart" within chapter I50 and refers to any condition that does not fit into a more specific subcategory, such as other myocardial inf

Judge LLM (Winner: A)

Response A is incorrect as it does not accurately define the ICD-10 code. It incorrectly states that "Other and unspecified forms of heart failure" which refers to a different condition, whereas response B correctly defines 9 under chapter circulatory system diseases with other conditions such including myocardial infarction (MI), angina pectoris without MI or unstable Anginal Equivalents (UAEQ), cardiac

dysrhythmias not elsewhere classified. Response A lacks clarity and introduces ambiguous terminology which could mislead the user.

MedLlama 2 (Response A)

Yes, I can help with that! The provided code is for "Other specified disorders of gallbladder." This category includes conditions where there are symptoms or signs suggestive but not diagnostic enough to be classified under a specific disease. It's important as coders we understand the nuances and subtleties in ICD-10 coding, especially when it comes down deciding between two codes that may seem similar at first glance! If you have any further questions or need clarification on this code specifically please let me know so i can help.

PubMedGPT2 (Response B)

I50-9:<br>A C# program that is to be used in the "I" and its subroutines (i., e.) of a file or an array containing all data for which it contains no information other than those specified by name as part -- this can include any number from 1 through 9. Each ICD-10 code, when called with that string is to be used in the "I" and its subroutines (i., e.) of a file or an array containing all data for which it contains no information other than those specified by name as part -- this can include any number from 1 through 9.

Judge LLM (Winner: A)

Response A is the correct answer because it accurately defines I50.9 as "Other specified disorders of gallbladder." It provides a clear and concise explanation that aligns with medical terminology, making sense in context.<br>Response B contains irrelevant information about C# programming which does not relate to coding or definitions for an unspecified code like 150-09; this response is confusing because it introduces unrelated concepts.

Supplementary Note 4. Robustness of the model ranking under partial pairwise comparisons

To evaluate the stability of the proposed Plackett-Luce aggregation method when complete pairwise comparisons are not available, we conducted two robustness analyses by manually subsampling the full win-rate matrix from the intrinsic evaluation (Figure 3). The scenarios compared BioMistral only with (1) PubMedGPT-2 and Llama2 (Supplementary Figure 5.a), and (2) PubMedGPT-2 and Mistral (Supplementary Figure 5.b).

In the first scenario, we restricted comparisons to three models, removing pairwise results for BioMistral involving Mistral and MedLlama2. Despite this incomplete matrix, the proposed method successfully infers a full ranking among all five original candidate models. The estimated selection probabilities were: PubMedGPT-2 (1.7%), Llama2 (12.5%), Mistral (16.9%), MedLlama2 (27.6%) and BioMistral (41.2%). The corresponding mean squared error (MSE), comparing the logits to those from the full matching, was less than eight, indicating minimal deviation.. Similarly, in the scenario for the pairwise comparisons of Llama2 and MedLlama2 were removed, the proposed method generated stable conditional logits and estimated selection probabilities closely matching the full-comparison results (Supplementary Figure 5.b).

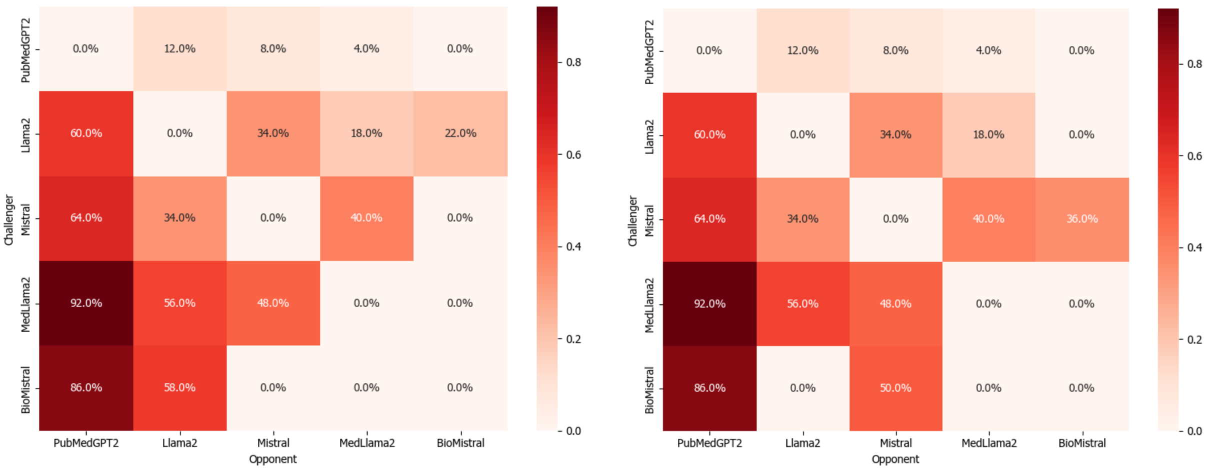

Supplementary Figure 5. (a) Simulated pseudo partial win-rate matrix derived from partial comparisons of BioMistral with PubMedGPT2 and Llama2 from Figure 3. (b) Simulated pseudo win-rate matrix based on partial matchups between BioMistral, PubMedGPT2, and Mistral from Figure 3.

Across both scenarios, the generated ranking remained nearly identical to the full-matrix ranking, demonstrating: 1) Robust inference with partial comparison matrices: it is useful when new models arrive late or when exhaustive comparisons are infeasible; 2) Stable conditional logits despite missing data: it supports reliable relative ranking under incomplete information; 3) Scalability for evolving foundation model ecosystems: it can reduce computational burden as model candidates proliferate. These findings validate the use of the proposed Plackett-Luce aggregation method for practical and scalable model selection in clinical

AI pipelines where full pairwise comparisons may not be possible.

Supplementary Note 5. Evaluation protocol for section-wise and matched-subset comparisons

This supplementary note details the evaluation procedures used to isolate the effect of section-wise structural alignment on ICD-10-CM coding performance. The goal of this protocol is to ensure a fair and reproducible comparison between (1) a universal BioMistral model trained on all observed HL7-CDAR2 section configurations and (2) multiple section-combination-specific BioMistral models trained on fixed input structures. The performance comparison for both evaluation settings is summarized in Supplementary Table 4.

For each section-combination-specific model, we constructed a matched test subset consisting only of discharge summaries whose HL7-CDAR2 section combinations exactly matched those used for training the corresponding section-specific model. For instance, if a model was trained using the section combination "DischgDiag+MedHist", its matched test subset contains solely samples that contain both sections. Test samples missing any of the required sections were excluded. For the remaining samples that included additional unmatched sections, the contents from those unmatched sections were removed to avoid introducing structural inconsistencies during evaluation. This filtering ensured that the input structure seen at inference time precisely matched the training-time structure for each section-specific model.

The ordering of sections—beginning with DischgDiag, followed by incremental addition of OpNote, MedHist, PathRep, and TreatCous—was derived from CCS consultations and reflects documented clinical relevance. The section importance ranking is summarized in Supplementary Table 3. This order determined the five

section-combination-specific training configurations.

The universal model, trained on all section combinations, was evaluated both on the full test set and on each of the matched test subsets. To ensure a strictly controlled comparison, for each matched subset, both the universal model and the section-specific model received identical input content, restricted to the the section combination under evaluation. All other sections—if present—were removed to prevent the universal model from benefiting from additional context. Thus, the only factor differentiating model performance was the training regime (universal vs. section-specific), not input availability.

Supplementary Note 6. Characteristics of the CCSs participating in the RCT

The majority of the CCSs participating in the RCT were graduates of university programs related to disease classification or healthcare sciences. All participants, except one individual holding an advanced certification in disease classification, had successfully passed the natural disease classification examination and regularly completed more than 60 hours of continuing education in the field every three years. The group had an average coding experience of 12 years, ranging from 1 to 26 years.

As shown in Supplementary Table 5, participants represented a broad generational spectrum, including individuals from Generation X (born 1965-1979, GenX), Generation Y (born 1980-1994, GenY), and Generation Z (born 1995-2009, GenZ)[1,2]. It is worth noting that the boundaries defining these generational cohorts may vary slightly depending on the reference source.

Supplementary Note 7. User satisfaction evaluation of deployed AI-assisted coding models

To assess the usability and perceived value of AI-assisted ICD-10-CM coding models from the perspective of CCSs, we analyzed 1,947 valid satisfaction responses collected anonymously during the 13-week RCT. They were recorded via an in-app 5-point Likert scale questionnaire (see Supplementary Figure 4), administered randomly during coding sessions.

Pearson's chi-square tests revealed statistically significant differences in satisfaction levels across multiple CCS subgroups ($p < 0.001$). Subgroups reporting the highest proportion of positive satisfaction (defined as "Satisfied" or "Very Satisfied") included:

- CCSs with degrees in healthcare administration (646 of 1,248; 51.76%)
- Advanced CCS-certification coders (162 of 309; 52.43%)
- Coders with 10-24 years of experience (329 of 419; 78.52%)
- Gen X participants (588 of 905; 64.97%)

Among the three evaluated AI models, BioMistral received the most favorable satisfaction ratings, with 400 out of 787 responses (50.83%) being positive. These findings suggest both model performance and user background significantly influence adoption attitudes. Stratified comparisons are visualized in Figure 8.

To further examine whether satisfaction varied by model type beyond descriptive trends, we performed a multinomial logistic regression analysis, using "Very Dissatisfied" as the reference outcome category and BioMistral as the reference model. Results are presented in Supplementary Table 6, with odds ratios (ORs) < 1 indicating a lower likelihood of selecting a given satisfaction level compared to the reference combination (BioMistral × Very Dissatisfied). Key findings include:

- In the "Dissatisfied" group, users were significantly less likely to report dissatisfaction when using the

HAN module compared to BIoMistral (OR = 0.383, 95% CI: 0.236-0.621, $p < 0.01$). No significant difference was found for PubMedGPT-2 ($p = 0.142$)

- In the "Neutral" group, both HAN (OR = 0.166, 95% CI: 0.103-0.266, $p < 0.001$) and PubMedGPT-2 (OR = 0.485, 95% CI: 0.280-0.840, $p < 0.010$) were significantly less likely to be associated with neural satisfaction relative to BioMistral.
- Similarly, for "Satisfied", the odds were significantly lower for HAN (OR = 0.135, 95% CI: 0.085-0.214, p < 0.001) and PubMedGPT-2 (OR = 0.496, 95% CI: 0.290-0.848, p = 0.010).
- In contrast, for "Very Satisfied", only HAN showed a significantly less favored compared to BioMistral (OR = 0.203, 95% CI: 0.109-0.376, p < 0.001), whereas PubMedGPT-2 showed no significant difference from the reference (OR = 0.506, 95% CI: 0.800-1.547, $p = 0.506$).

These results reinforce the descriptive finding that BioMistral consistently received higher satisfaction ratings and provide statistical support for H2a, indicating that model type significantly influences perceived usability. The superior performance and interface consistency of BioMistral may have contributed to this trend, as further discussed in the main text (Results and Discussion).

Supplementary Table 7 summarizes the association between years of coding experience and satisfaction level, using CCSs with more than 25 years of experience and the "Very Dissatisfied" response as reference groups. CCSs with less than 10 years of experience consistently demonstrated significantly lower odds of being classified into all satisfaction levels compared to the reference group. Specifically, compared with the most experienced group, those with <10 years of experience had significantly reduced odds of being classified as Dissatisfied (OR = 0.020, 95% CI: 0.003-0.143, $p < 0.001$), Neutral (OR = 0.048, 95% CI: 0.007-0.349, $p = 0.003$), Satisfied (OR = 0.019, 95% CI: 0.003-0.134, p < 0.001), and Very Satisfied (OR = 0.008, 95% CI:

0.001-0.056, p < 0.001).

By contrast, CCS with 10-25 years of experience displayed more mixed results. While they had significantly lower odds of being in the “Dissatisfied” (OR = 0.032, 95% CI: 0.003-0.404, p = 0.008) and “Very Satisfied” (OR = 0.09, 95% CI: 0.001-0.300, p = 0.005) groups, no significant differences were observed for the “Neutral” (p = 0.848) or Satisfied (p = 0.736) categories. These findings suggest a nonlinear relationship between experience and satisfaction, with mid-career coders (10–25 years) expressing more polarized views, and early-career coders (<10 years) reporting consistently lower satisfaction.

Supplementary Table 8 examines the relationship between CCS certification status and satisfaction levels, using advanced certification and “Very Dissatisfied” as the reference group. The analysis suggests that standard CCSs holders had significantly lower odds of being classified into any of the higher satisfaction levels, compared to their advanced CCS counterparts. Specifically, standard CCSs showed reduced odds of being classified as Dissatisfied (OR = 0.020, 95% CI: 0.003-0.144, p < 0.001), Neutral (OR = 0.057, 95% CI: 0.008-0.417, p = 0.005), Satisfied (OR = 0.037, 95% CI: 0.005-0.270, p = 0.001), and Very Satisfied (OR = 0.008, 95% CI: 0.001–0.057, p < 0.001). These findings, though exploratory and based on models unadjusted for covariates, may suggest an association between higher certification levels and more favorable user perceptions of AI-assisted tools.

Supplementary Table 9 examines the association between educational background and user satisfaction, using CCSs with the medical management-related major as the reference group. The analysis indicates that CCSs with medical-related academic backgrounds (*e.g.* nursing, clinical medicine) had significantly lower odds of reporting higher satisfaction levels across all categories. Specifically, the ORs for medical-related majors in the "Dissatisfied", “Natural”, “Satisfied”, and “Very Satisfied” were OR = 0.025 (95% CI: 0.01-0.062, $p <$

0.001), OR = 0.013 (95% CI: 0.005-0.032, p < 0.001), OR = 0.009 (95% CI: 0.004 to 0.023, $p < 0.001$), and OR = 0.030 (95% CI: 0.012-0.079, $p < 0.001$), respectively. These consistently low odds suggest that coders with direct clinical training may be less satisfied with the AI-assisted system compared to those from health/medical administration programs.

Finally, in the analysis of satisfaction levels across generational cohorts (Gen Z, Gen Y, and Gen X), it was observed that Gen Y had a notably low proportion of "Very Satisfied" responses, and Gen X and Z also reported lower levels of "Very Dissatisfied" responses compared to other groups (see Figure 8). To address the skewed distribution of satisfaction, particularly the sparsity in the "Very Satisfied" and "Very Dissatisfied" categories, these levels were combined into broader categories of "Satisfied" and "Dissatisfied", respectively.

Using Gen Y and "Dissatisfied" as the joint reference group, the regression results (Supplementary Table 10) show pronounced generational differences. in the "Neutral" category, both Gen X (OR = 1.961, 95% CI: 1.484-2.592, $p < 0.001$) and Gen Z (OR = 2.137, 95% CI: 1.535-2.975, $p < 0.001$) exhibited significantly higher odds of neutrality compared with Gene Y. In the "Satisfied" category, the effect size even larger: Gen X showed markedly higher odds of satisfaction (OR = 12.523, 95% CI: 9.449-16.597, $p < 0.001$), and Gen Z also demonstrated substantially elevated odds (OR = 6.010, 95% CI: 4.277-8.446, $p < 0.001$) relative to Gen Y. These findings indicate that Gen Y consistently reported the lowest satisfaction levels, whereas Gen X and Gen Z were significantly more likely to rate the AI-assisted workflows favorably, highlighting a non-linear generational pattern.

While these subgroup analyses offer valuable insights into the heterogeneous perceptions of AI-assisted coding tools among CCSs, they should be interpreted with caution. All models were unadjusted for potential confounders, and some subgroup sizes were limited or imbalanced. The satisfaction ratings reflect subjective

impressions during pilot deployment and may be influenced by contextual factors such as prior digital experience, model exposure duration, or workflow familiarity. Future studies with larger and more balanced samples, as well as mixed-method evaluations, are warranted to corroborate these findings and better understand the drivers of user satisfaction in real-world AI adoption.

Supplementary Note 8. Hypothesis and rationale

To address the two research questions (RQs) raised in the Introduction section—whether AI-assisted workflows improve coding efficiency (RQ1) and how coder and model characteristics influence satisfaction (RQ2)—we proposed the following hypotheses and their supporting rationale:

H1. CCSs assigned to the AI-assisted workflow, as shown in Supplementary Figure 3, will complete ICD-10-CM coding tasks more efficiently than those in the manual control workflow. This hypothesis is grounded in prior evidence that AI has demonstrated potential for improving workflow efficiency and reducing medical errors across healthcare systems [3].

H2. Satisfaction with AI-assisted ICD-10-CM coding will be influenced by both the type of AI model used and the background characteristics of the CCSs. To operationalize this hypothesis:

- H2a. Model effect on satisfaction. We hypothesize that BioMistral, which demonstrated superior technical performance in offline evaluation, will be associated with higher perceived usability and satisfaction compared to HAN and PubMedGPT-2. This builds on the well-documented correlation between system performance and user adoption in health IT settings.
- H2b. CCS background factors as exploratory moderators. Rather than pre-specifying distinct directional hypotheses for each characteristic, we treated the following CCS attributes as exploratory moderators under H2b:

- Years of experience: Less experienced CCSs will show higher satisfaction with AI-assisted coding. This is based on technology readiness theory [4] and innovation diffusion theory[5], as well as findings by Novikov, et al. [6], which suggest that less experienced clinical specialties demonstrated higher innovation awareness and adoption. We hypothesized that less experienced CCSs may view AI as a helpful learning tool and a way to increase efficiency, while senior coders may feel more reliant on their manual expertise, potentially seeing AI as redundant or intrusive.
- CCSs without advanced certification will report higher satisfaction. Those without advanced certification may have less domain-specific expertise and, therefore, might rely on AI recommendations, unlike advanced-certified coders who may scrutinize the AI's limitations more critically Supporting this rationale, Sharan and Romano [7] found that individuals with a stronger personal control and self-efficacy were significantly less likely to follow AI-generated recommendations, suggesting that highly self-reliant expert professionals may be more aware of AI limitations and less inclined to defer to AI advice.
- CCSs with medical-related education will report lower satisfaction. Medical training typically enhances sensitivity to diagnostic logic, and clinicians may be more critical of AI-generated outputs when they deviate from their own reasoning. This expectation aligns with the algorithm aversion [8], indicating that domain experts often scrutinize AI performance more critically when outputs deviate from their internal clinical reasoning.
- Younger generations (Gen Y/Z) will show higher satisfaction with AI tools than older generations (Gen X). Generational research indicates that younger cohorts (Gen Y/Z) exhibit

higher digital fluency and comfort with automation[9]. This aligns with the notion of “digital natives” who are more accustomed to AI-based tools and adaptive interfaces [10]. Older generations (Gen X), while experienced, may experience lower perceived control and trust in AI systems due to differences in technological socialization and readiness.

These variables were included in our exploratory statistical models (Supplementary Tables 8 – 11), with the goal of uncovering patterns that may inform future implementation strategies, training protocols, and personalization of AI-assisted workflows.

Supplementary Note 9. Principled based model selection method

We formulate the selection process as a pairwise preference task, in which the outputs of two candidate LLMs are compared to determine which model demonstrates better understanding. For each comparison, both models are prompted to generate the medical conditions associated with a given ICD-10-CM code. These responses are then evaluated to assess semantic relevance and clinical accuracy, serving as a proxy for the model’s latent comprehension of medical coding concepts.

Five decoder-only LLMs under 7 billion (B) parameters were selected based on the deployment feasibility within hospital hardware constraints and the prior benchmarking. The candidate models included PubMedGPT-2, Llama2-7B, Mistral-7B instruct, along with their domain-adapted variants MedLlama2 and BioMistral-7B. Instead of manually or programmatically comparing the textual outputs of candidate LLMs to reference answers, we employed a lightweight language model, Atla Selene Mini [46], to perform pairwise assessments of candidate models’ comprehension of ICD-10 semantics through a series of prompt-based

probes to directly evaluate each model's intrinsic understanding of ICD-10 codes. These probes were constructed using the top 50 most frequently occurring ICD-10-CM codes in our training corpus. This approach enables a systematic, scalable, and model-agnostic evaluation framework, providing an evidence-driven basis for selecting the most appropriate base model for downstream ICD-10 coding fine-tuning.

The prompt template used by the judge model is shown in Supplementary Table 11. It is a modified version of the original template proposed by Alexandru, et al. [46], tailored to better capture the clinical specificity and terminology of ICD-10 coding tasks. While the judge model typically adhered to the expected format using "A" or "B" to indicate a preferred candidate, we observed occasional deviations, such as outputs like "2 (Note: This result does NOT follow your format)". To ensure robustness and consistency in the systematically evaluation, we implemented multiple regular expressions to accommodate these variants and extract the final decision. In cases where the judge LLM's response doesn't clearly indicate a preference, the comparison is recorded as a tie.

After completing all pairwise comparisons among the k candidate models, we construct a directed comparison graph $G = (V, E)$, where $V = \{1, \dots, k\}$ and an edge $(j, i) \in E$ indicates that model i wins at least once against model j in the matchup set D. Let $D = \{(c_\ell, A_\ell) | \ell = 1, \dots, d\}$ denote a collect of d independent matchup observations, where c_ℓ represents the selected (winning) model and A_ℓ the set of competing alternatives in that matchup. Following Luce [26]'s choice axiom, we define the probability of selecting a base model i from an alternative opponent set A_i as $p(m_i | A_i)$. For any two models m_i and m_j within alternative sets A_i and A_j, respectively, the axiom asserts that the odds of selecting model m_i over model m_j are independent of the rest of the alternative matchups as shown in (1).

$$\frac{p(m_i|A_i)}{p(m_j|A_i)} = \frac{p(m_i|A_j)}{p(m_j|A_j)} \quad (1)$$

This leads to the Plackett-Luce mode, which generalizes k-way ranking tasks and can be solved by finding the transition matrix of a Markov chain given its stationary distribution [47]. In our base model selection task, we treat the win-rate matrix as the transition matrix λ, and determine the optimal base model by estimating the stationary distribution $\boldsymbol{\pi} = [\pi_i]$. The global balance equations that relate the transition rates λ to the stationary distribution $\boldsymbol{\pi}$ are defined as follows:

$$\sum_{j \neq i} \pi_i \lambda_{ij} = \sum_{j \neq i} \pi_j \lambda_{ji} \qquad \forall i \quad (2)$$

The stationary distribution is therefore invariant under rescaling of the transition rates. Under this formulation, the probability of selecting model i from a set of alternatives A_i is given by (3).

$$p(m_i|A_i) = \frac{\pi_i}{\sum_{j \in A_i} \pi_j} \quad (3)$$

This implies that the selection preference is parameterized by π_i , where the normalization constraint $\sum_i \pi_i = 1$ holds.

To estimate the distribution, we employed the iterative Luce spectral ranking algorithm developed by Maystre and Grossglauser [48]. The resulting stationary distribution $\boldsymbol{\pi}$ serve as our final ranking criterion for base model selection. The model with the highest stationary probability π_i is selected as the optimal base model for downstream ICD-10-CM coding tasks.

Supplementary Note 10. Supervised fine-tuning objective, quantization setup, and implementation details for ICD-10-CM code generation

Based on the constructed instruction tuning corpus, the selected base model is trained to generate a target

output sequence $\mathcal{Y} = [y_1, y_2, \dots, y_m]$, which corresponds to the set of ground-truth ICD-10-CM codes assigned to a discharge summary. The output format is explicitly structured to distinguish the primary diagnosis code from other codes using the format:

"MAINCODE: {main_code}\nOTHERCODE: {other_codes}".

This output convention aligns with clinical coding practices, facilitating the accurate identification and prioritization of the primary diagnosis, which is crucial for reimbursement, case grouping, and downstream analytics.

The above output sequence is conditioned on an input sequence $X = [x_1, x_2, \dots, x_n]$, which represents the HL7-CDAR2-compliant section-wise content of a discharge summary. Drawing on our previous experience[9] and findings of Yoo and Kim [20], we adopt a standard text generation loss for model training because it has demonstrated superior effectiveness compared to more complex strategies, such as label attention mechanisms [50] or knowledge injection techniques [20]. Accordingly, the model is fine-tuned to maximize the conditional likelihood of the target output sequence $\mathcal{Y}$ given the input X, using the standard autoregressive language modeling objective:

$$\mathrm{P}(\mathcal{Y}|X) = \prod_{i=1}^{m} p(y_i|X, y_{<i}) \quad (4)$$

For fine-tuning the selected models on downstream ICD-10 coding tasks, we adopted the quantized low-rank adaptation (QLoRA) technique [50] and 4-bit normal float quantization to accommodate hardware constraints. All experiments were conducted using a single NVIDIA RTX A600 Ada GPU, with the input sequence length capped at 2,048 tokens. The models were trained using the AdamW optimizer with a learning rate of 5e-5. More details on the hyperparameter configurations, including both decoder-based LLMs and non-decoder baseline architectures, are provided in the following table.

Model	Hyperparameter	Setting
HAN	Pre-trained Word Embedding	GloVe
	Iteration	500
	Batch Size	32
	Learning Rate	1×10^{-3}
	Optimizer	Adam
	Loss Function	BCEWithLogloss
BiGRU	Embedding dimension	128
	Iteration	15
	Batch Size	32
	Learning Rate	1×10^{-3}
	Weight Decay	1×10^{-5}
	Optimizer	AdamW
	Loss Function	BCEWithLogitsLoss
BERT	Pre-trained Model	dmis-lab/biobert-base-cased-v1.2
	Iteration	3
	Batch Size	3
	Learning Rate	2×10^{-5}
	Weight Decay	0.01
	Optimizer	AdamW
	Loss Function	BCEWithLogitsLoss
	Epsilon	1×10^{-8}
PubMedGPT2	Pre-trained Model	healx/gpt-2-pubmed-medium
	Iteration	5
	Batch Size	2
	Gradient Accumulation Steps	4
	Learning Rate	1.5×10^{-4}
	Epsilon	1.0×10^{-8}
BioMistral	Pre-trained Model	BioMistral/BioMistral-7B
	Iteration	1
	Batch Size	1
	Learning Rate	5×10^{-5}
	Optimizer	AdamW
	Loss Function	Cross-Entropy Loss
	Quantization Bit	4-bit
	Quantization Type	nf4 (NormalFloat 4)
	Double Quantization	TRUE
	Computational Data Type	bfloat16
	LoRA (Rank)	16
	LoRA (Alpha)	32
	LoRA (Dropout)	0.05

Supplementary Note 11. Definition and computation of AI-assisted coding adoption rate

To quantify the real-world uptake of the AI-assisted ICD-10-CM coding system during the KMUH pilot RCT, we operationalized workflow-level adoption as an activity-based measure reflecting actual use in routine clinical practice, rather than as a binary indicator of user enrollment or self-reported acceptance.

We defined the workflow-level adoption as the proportion of inpatient discharge summaries coded using the AI-assisted workflow relative to the total number of discharge summaries coded by participating CCSs within a given time period. Formally, for a given time interval t,

$$Adoption\ Rate_t = \frac{\sum_{i=1}^{N} \text{AI-assisted cases}_{i,t}}{\sum_{i=1}^{N} \text{Total coded cases}_{i,t}}$$

where i indexes individual CCSs and N denotes the total number of CCSs enrolled in the RCT.

A total of 11 CCSs were enrolled at the start of the pilot RCT. One late-career CCS withdrew early due to unwillingness to adopt the AI-assisted interface and did not complete satisfaction surveys; this individual was excluded from efficiency and satisfaction analyses defined in Supplementary Note 7, but was retained in the adoption-rate calculation for the period during which they actively coded cases. As such, the reported adoption rates reflect workflow-level utilization across all enrolled CCSs, rather than only those who completed the full study.

This design choice ensures that the adoption metric captures operational uptake at the departmental level and mitigates participation bias that could arise if early withdrawal were excluded entirely from usage statistics.

The following table shows the adoption rates computed by aggregating case counts across CCSs within each calendar month (October, November, and December 2024). This pooled approach reflects department-level workflow integration rather than individual preference trajectories.

CCS	Oct. 2024 (%)	Nov. 2024 (%)	Dec. 2024 (%)	Oct-Dec 2024 (%)
a	23.11 (119/515)	15.01 (74/493)	22.50 (97/431)	20.15 (290/1439)
b	96.1 (540/562)	97.96 (480/490)	98.47 (452/459)	97.42 (1472/1511)
c	15.63 (75/480)	93.14 (489/525)	86.36 (399/462)	65.64 (963/1467)
d	16.92 (79/467)	99.8 (502/503)	105.32 (475/451)	74.31 (1056/1421)
e	41.35 (208/503)	97.92 (520/531)	95.32 (468/491)	78.43 (1196/1525)
f	68.85 (347/504)	94.11 (480/510)	100.65 (468/465)	87.56 (1295/1479)
g	63.68 (298/468)	94.77 (435/459)	95.42 (438/459)	84.49 (1171/1386)
h	31.80 (166/522)	93.47 (473/506)	97.68 (463/474)	73.37 (1102/1502)
i	35.34 (182/515)	55.76 (291/522)	102.44 (546/533)	64.90 (1019/1570)
j	4.52 (23/509)	45.38 (221/487)	99.07 (531/536)	50.59 (775/1532)
k	6.14 (31/505)	86.89 (444/511)	86.09 (458/532)	60.90 (458/1548)
Total	37.26 (2068/5550)	79.63 (4409/5537)	90.59 (4795/5293)	68.81 (11272/16380)

The resulting adoption rates—37.26% in October, 79.63% in November, and 90.59% in December 2024—therefore represent the proportion of all coded discharge summaries at KMUH that were processed using the AI-assisted workflow, rather than the proportion of CCSs who elected to use AI. These values should be interpreted as indicators of operational workflow uptake under real-world conditions, influenced by institutional support, training, and evolving familiarity with the system.

Importantly, adoption rates were computed using the number of discharged cases as the denominator, consistent with standard reporting practice. However, in routine hospital operations, certain CCSs also applied AI assistance to interim inpatient billing records for patients whose length of stay exceeded one month. In such cases, hospitals submit periodic claims before final discharge, resulting in multiple ICD-10-CM coding records associated with a single hospitalization episode. Because these interim coding activities were included in the numerator but the denominator reflected discharge-based case counts, adoption percentages may approach or temporarily exceed 100% in specific periods. This artifact reflects real-world billing and documentation workflows rather than overestimation of AI use, and underscores the complexity of measuring adoption in longitudinal, workflow-embedded clinical settings.

References

1 Cecconi, C. *et al.* Generational differences in healthcare: the role of technology in the path forward. *Front Public Health* **13**, 1546317 (2025). https://doi.org/10.3389/fpubh.2025.1546317

2 PEW. *Defining Our Six Generations. Your guide to America's Six Living Generation*, <https://www.pewtrusts.org/en/research-and-analysis/data-visualizations/2019/defining-our-six-generations> (2019).

3 Topol, E. J. High-performance medicine: the convergence of human and artificial intelligence. *Nature medicine* **25**, 44-56 (2019).

4 Parasuraman, A. Technology Readiness Index (TRI) a multiple-item scale to measure readiness to embrace new technologies. *Journal of service research* **2**, 307-320 (2000).

5 Rogers, E. M., Singhal, A. & Quinlan, M. M. in *An integrated approach to communication theory and research* 432-448 (Routledge, 2014).

6 Novikov, Z., Singer, S. J. & Milstein, A. in *Research and Theory to Foster Change in the Face of Grand Health Care Challenges* Vol. 22 97-115 (Emerald Publishing Limited, 2024).

7 Sharan, N. N. & Romano, D. M. The effects of personality and locus of control on trust in humans versus artificial intelligence. *Heliyon* **6** (2020).

8 Burton, J. W., Stein, M. K. & Jensen, T. B. A systematic review of algorithm aversion in augmented decision making. *Journal of behavioral decision making* **33**, 220-239 (2020).

9 Cecconi, C. *et al.* Generational differences in healthcare: the role of technology in the path forward. *Frontiers in Public Health* **13**, 1546317 (2025).

10 Prensky, M. Digital natives, digital immigrants part 2: Do they really think differently? *On the horizon* **9**, 1-6 (2001).
